# Interpretable manifold learning for T-wave alternans assessment with electrocardiographic imaging

**DOI:** 10.1016/j.engappai.2024.109996

**Published:** 2025-01-17

**Authors:** E. Sánchez-Carballo, F.M. Melgarejo-Meseguer, R. Vijayakumar, J.J. Sánchez-Muñoz, A. García-Alberola, Y. Rudy, J.L. Rojo-Álvarez

**Affiliations:** a Universidad Rey Juan Carlos, Department of Signal Theory and Communications, Telematics and Computing, Cam. del Molino, 5, Fuenlabrada, 28942, Madrid, Spain; b Washington University in St. Louis, Cardiac Bioelectricity and Arrhythmia Center, 1 Brookings Drive, St. Louis, 63130-4899, MO, United States; c Hospital Clínico Universitario Virgen de la Arrixaca, Arrhythmia Unit, Ctra. Madrid-Cartagena, s/n, El Palmar, 30120, Murcia, Spain; d D!lemma Ltd startup, Cam. del Molino, 5, Fuenlabrada, 28942, Madrid, Spain

**Keywords:** T-wave alternans, Sudden cardiac death, Electrocardiographic imaging, Interpretable manifold learning, Explainable artificial intelligence, Electrophysiological biomarkers

## Abstract

T-wave alternans (TWA) is a biomarker for sudden cardiac death prediction, characterized by subtle variations in the amplitude or morphology of consecutive T-waves in electrocardiographic studies. Electrocardiographic imaging (ECGI) offers increased spatial resolution, enabling TWA distribution analysis across the epicardium. However, existing TWA estimation methods disregard ECGI spatial information by analyzing each signal independently. To address this gap, we present a novel, subject-specific, interpretable manifold learning-based TWA estimation method tailored to ECGI. First, Uniform Manifold Approximation and Projection (UMAP) reduces input data dimensions. Second, the Louvain algorithm detects communities and identifies the TWA-dominant community. Finally, the location of this community and the rest of the communities is compared, and a Bootstrap-based TWA classifier is applied. A customized Shapley additive explanations method was developed to identify the signal segments most affecting the algorithm decisions to enhance explainability. Reducing the input data to 18 dimensions improved the separation of the TWA-dominant community, with an average normalized distance of 0.28. The Bootstrap analysis showed that the TWA-dominant community had a distance metric up to 0.2 above the confidence interval upper limit. The TWA-dominant community input signals showed different TWA patterns, namely, hump-shaped and amplitude-shifted TWA, and the interpretability algorithm revealed that UMAP focuses on them when projecting points into the latent space. Our method achieved maximum accuracy in subjects with known outcomes and made consistent patient decisions based on input signals. This study introduces the first ECGI-specific TWA detection method. Its subject-specific nature enables the extraction of individual-specific characteristics, offering personalized diagnostic insights.

## 1. Introduction

Sudden cardiac death (SCD) is a public health problem, as it is one of the leading causes of mortality worldwide (Roth et al., 2017; Yow et al., 2024). Reducing its incidence should be a priority and demands an in-depth study of preemptive measures. One pathway worth exploring is the identification of patients at high risk of experiencing SCD, which can be achieved using predictors of SCD risk, such as T-wave alternans (TWA). TWA measures changes in the amplitude or morphology of consecutive T-waves in electrocardiographic (ECG) studies, and it has been demonstrated to be a successful and reliable stratification marker for malignant ventricular arrhythmias (Armoundas et al., 2002; Narayan, 2006; Cutler and Rosenbaum, 2009). There are specific illnesses that are more associated with the appearance of TWA, including ischemic cardiomyopathy (ICM) (Ikeda et al., 2002; Chow et al., 2006; Janusek et al., 2015), Prinzmetal angina (Kleinfeld and Rozanski, 1977; Rozanski and Kleinfeld, 1982; Verrier and Nearing, 1994), and Long QT syndrome (LQTS) (Platt et al., 1996; Gadage, 2018; Yang et al., 2022). However, the detection and estimation of TWA are not without limitations, which leads to its limited use in everyday medical procedures (Aro et al., 2016). One such limitation is that the difference between consecutive T-waves is usually in the order of microvolts. Therefore, TWA cannot be visually assessed by a physician and requires using signal processing, estimation, and visualization techniques (Richter et al., 2005; Hohnloser et al., 2009). Due to the high prevalence of SCD, the importance of improving existing TWA estimation methods and developing new ones remains high.

Multiple works highlight the importance of performing spatiotemporal studies when studying heart diseases in general (Rudy and Burnes, 1999; Lara-Hernandez et al., 2021), and TWA in particular (Trayanova et al., 2021; Behr and Ensam, 2016; Caulier-Cisterna et al., 2020a,b). This is because arrhythmic events usually occur in specific areas of the heart, often due to the incorrect behavior of a cluster of cells or the presence of a scar (Němec et al., 2016; Nelson et al., 2019). In this context, electrocardiographic imaging (ECGI) is helpful for cardiac research and clinical care, as it is a novel imaging technique that noninvasively maps the electrical activity of the heart on the cardiac muscle surface from torso ECG recordings and geometrical information from computed tomography (CT) or magnetic resonance imaging (MRI) scans (Ramanathan et al., 2004, 2006; Rudy, 2017).

ECGI offers the opportunity to analyze heart health with spatial accuracy. This novel imaging modality can be an alternative to conventional ECG to detect malignant arrhythmias that can lead to SCD (Lou et al., 2023). While a traditional ECG study only provides an approximate view of the heart electrical activity, ECGI provides a more detailed version with considerably increased spatial resolution. Currently, electrical instability is not directly diagnosed with conventional ECG studies. Instead, suspicions of electrical instability usually arise from other specific patient characteristics, such as the underlying cardiological disease, the frequency of arrhythmias, symptoms of sporadic arrhythmia like syncope, and Holter recordings showing complex ventricular ectopy. In these cases, doctors design the plan to follow depending on the severity of the situation. If there is a high risk of SCD, as in the case of Brugada syndrome with syncope, doctors directly indicate the need for an implantable cardioverter defibrillator. If the risk of SCD is intermediate, as in the case of myocardiopathies, doctors suggest additional medical tests such as genetic studies, Holter monitoring, or MRI for detecting fibrosis (Cluitmans et al., 2015; Bariani et al., 2021). When an invasive test is required, it is usually an electrophysiological study, where catheters are inserted into the heart through blood vessels, or coronary angiography, which involves injecting a special dye visible by X-ray into the coronary arteries through a catheter. These two invasive studies enable cardiologists to localize the source of arrhythmias. Although both are generally safe, they have risks, including bleeding, infection, blood vessel damage, and arrhythmias (Scanlon et al., 1999; Muresan et al., 2019). With the arrival of ECGI, the information that was previously obtained through invasive procedures can now be gathered non-invasively, allowing the analysis of the heart from a spatiotemporal perspective and facilitating more effective diagnosis of arrhythmias and more appropriate drug or ablation therapy (Pereira et al., 2020; Parreira et al., 2023). Results from recent investigations have demonstrated not only the superiority of ECGI compared to conventional ECG for arrhythmia detection (Graham et al., 2020), but also its effectiveness comparable to that of invasive mapping methodologies (Ghadban et al., 2020).

In recent years, numerous methods have been developed to estimate TWA, an essential marker of cardiac electrical instability, from ECG signals. The most commonly used TWA estimation methods in the literature are the temporal, spectral, and modified moving average methods (Barquero-Pérez et al., 2009; Gimeno-Blanes et al., 2016). These TWA estimation procedures have been successfully employed in ambulatory ECG studies (Verrier and Nearing, 2003; Lewek et al., 2016), and they function by comparing the even and odd T-waves of a single ECG signal. However, a significant gap in the current knowledge is that these methods are primarily designed for traditional ECG signals and are not specifically tailored to take advantage of the diagnostic possibilities that current ECGI systems can offer clinicians. ECGI provides spatial and temporal information by recording multiple ECG signals across the body surface. When traditional TWA estimation methods are applied to ECGI data, they analyze each signal independently, which is time-consuming and inefficient. Additionally, these methods focus exclusively on temporal variations, failing to leverage the spatial information inherent in ECGI fully. This limitation reduces the ECGI potential to improve TWA detection accuracy. Therefore, there are few scientific works focused on the analysis of TWA using ECGI (Coll-Font et al., 2018; Rojo-Álvarez et al., 2018; Caulier-Cisterna et al., 2020b), which makes it necessary to develop new TWA estimation methods that can leverage both the spatial and temporal information provided by ECGI simultaneously and efficiently.

To address this gap, we have developed an explainable TWA estimation method based on manifold learning (MnL), specifically designed to be subject-specific and to work with ECGI data. MnL is a sub-field of machine learning (ML) that aims to reduce input data dimensions by projecting the original data into a lower-dimensional space while maintaining the original data structure as much as possible (Ma and Fu, 2012). Previous studies have demonstrated the adequacy of ML for cardiac disease diagnosis and prediction (Mathur et al., 2020; Rubini et al., 2021; Ogunpola et al., 2023). More specifically, recent studies using ML algorithms for TWA analysis have emerged (Pascual-Sánchez et al., 2024; Nearing and Verrier, 2024), motivating the proposal to use explainable MnL algorithms for TWA analysis. Unlike traditional approaches, which analyze each ECG signal independently, MnL allows us to process both the temporal and spatial information offered by ECGI at the same time. This enables our proposed method to fully utilize the multidimensional data provided by ECGI fully, enhancing the precision of TWA estimation and localizing areas of the cardiac muscle with the most pronounced TWA. While ML-based TWA estimation methods have gained popularity recently, they typically focus on training models to recognize TWA patterns across different individuals, often requiring large labeled datasets. In contrast, our approach is subject-specific and specifically designed to analyze the unique features of each individual ECGI data, ensuring a personalized analysis. It applies unsupervised learning to detect TWA within an individual by differentiating between signals with and without alternans. This subject-specific approach allows for more accurate and relevant identification of TWA, which can vary significantly among individuals, leading to more precise and personalized diagnostic and treatment outcomes.

Uniform Manifold Approximation and Projection (UMAP) (McInnes et al., 2018) is a MnL algorithm that reduces the dimensions of input data while preserving its global and local structure. It is one of the state-of-the-art non-linear techniques for dimensionality reduction and data visualization. As TWA patterns are complex, we decided to use an embedding method like UMAP to capture not only the linear relationships, as traditional dimensionality reduction algorithms like principal components analysis (Jolliffe, 2002) do, but also the non-linear ones. Additionally, UMAP addresses some disadvantages of other non-linear MnL methods, such as t-distributed stochastic neighbor embedding (van der Maaten and Hinton, 2008). Its key advantages include the ability to preserve both global and local structures of the original data, its effectiveness in identifying the optimal clusterable manifold, its capacity to handle large datasets, and its lower computational cost, which sets it apart from other algorithms with similar objectives (Allaoui et al., 2020; Ghojogh et al., 2021; Oskolkov, 2022; Ghojogh et al., 2023). UMAP has recently been utilized in ECG studies primarily for visualizing and analyzing data (Khunte et al., 2023; Nogimori et al., 2024). In the embedded space generated by an MnL algorithm like UMAP, ECG signals from nearby epicardial locations are expected to cluster closely together, as they share similarities but differ more from signals located further away. If TWA exists in one ECG signal, it is likely to exist in similar ECG signals. One exciting approach to localizing these signal groups in a latent space could be through community detection algorithms (Gulbahce and Lehmann, 2008; Fortunato, 2010). These methods identify similar data points by considering characteristics such as topology, with algorithms like the Louvain algorithm (Blondel et al., 2008) being particularly noteworthy. This algorithm is known for its efficiency and scalability in detecting communities within large networks, capable of identifying both small and large communities effectively (Javed et al., 2018; You et al., 2020). Additionally, Bootstrap resampling emerges as a robust alternative among the methods that help derive inference and validate results. It is a statistical method used to estimate the distribution of a sample statistic by resampling the original data with replacement. It generates an empirical distribution of the statistic, enabling the calculation of confidence intervals (CIs) and supporting hypothesis testing (Grunkemeier and Wu, 2004; Dixon, 2006). Studies have demonstrated that Bootstrap can be successfully used for cardiac disease detection (Goya-Esteban et al., 2010; Alonso-Atienza et al., 2012), and specifically for TWA detection and estimation (Rojo-Álvarez et al., 2008). Its flexibility and robustness make it appropriate for validating any decision-making process, including those in the medical field.

One significant limitation of many ML models is their lack of interpretability, which complicates understanding how these models arrive at their decisions. In response to this challenge, various efforts have been made to enhance the interpretability of artificial intelligence (AI) algorithms. Among the developed solutions, Shapley Additive Explanations (SHAP) is a prominent algorithm designed to address this issue (Shapley, 1953). SHAP breaks down the predictions made by ML algorithms into contributions from individual variables. It leverages Shapley values from cooperative game theory to quantify how much each feature contributes to the model predictions. The main idea of SHAP is to treat each variable as a player in a game where the payout is the model prediction. Shapley values represent the average marginal contribution of a variable across all possible combinations of other variables, making SHAP mathematically consistent. One of SHAP key strengths is its ability to offer both global and local interpretability. Globally, SHAP provides an overall picture of which variables are most important, highlighting how certain variables impact the model decision-making process on average. Locally, SHAP allows the explanation of individual predictions, showing how much each variable contributes positively or negatively to the output. This is particularly useful in healthcare, where understanding why a model made a specific decision is crucial. By providing a unified feature importance measure, SHAP enables users to gain insights into which input variables most significantly influence model output, facilitating a deeper understanding of the decision-making process.

New advanced model-free, uncertain, deep AI-based optimal algorithms have been recently developed to enable early diagnoses by leveraging the inherent structure of complex data, see for example Tutsoy and Gul-Koç (2024). Similarly, our approach employs MnL techniques, which, like these AI-based methods, do not rely on predefined models but rather adapt to the data intrinsic geometry. This model-free aspect allows for more flexible and accurate detection of TWA. Our algorithm integrates community detection, which automatically identifies groups of signals with shared features, like AI systems that uncover hidden patterns within data. Finally, Bootstrap resampling introduces an uncertainty-handling component, as advanced AI algorithms incorporate uncertainty to enhance decision-making. This combination of MnL, community detection, and Bootstrap resampling positions our algorithm alongside the latest AI-based diagnostic tools, offering a robust, adaptive, and data-driven method for early SCD detection.

This research proposes the first explainable TWA detection method tailored to ECGI data. This method integrates UMAP for dimensionality reduction, the Louvain algorithm for community detection, and Bootstrap resampling to decide on the presence or absence of TWA. To enhance interpretability, a customized version of SHAP, suitable for ECG signals, is applied, allowing for the explanation of the decisions made by the proposed algorithm. The work is structured as follows. First, a literature review is provided in Section 2, where different studies related to ours are revised. Then, the methodology followed to develop the proposed method is explained in Section 3, including the database description, the ECGI acquisition process, the ECG signal processing steps, the components of the proposed algorithm, and the customized interpretability algorithm. Next, the experiments and results are detailed in Section 4. A discussion of the implications and limitations of this work is provided in Section 5, followed by the conclusions and future research directions presented in Section 6.

## 2. Literature review

TWA estimation has gained significant attention recently due to its established role as a reliable predictor of SCD. Despite its potential, accurately detecting TWA remains challenging, as the variations between consecutive T-waves are often subtle and easily mistaken for noise. However, the complexity of TWA estimation lies not only in the subtle nature of these variations but also in the careful consideration required throughout the entire process, from preprocessing and signal conditioning to the final analysis. Each step of the estimation process demands special attention to ensure that TWA patterns are accurately captured.

Multiple studies highlight the importance of preprocessing before analyzing general biomedical signals and ECG signals. Eliminating baseline noise and high-frequency noise from ECG signals is essential for drawing relevant conclusions in subsequent analyses. Many methods reported in the literature aim to remove as much noise as possible without compromising physiological information. In Rashid et al. (2011), the authors developed the projection pursuit gradient ascent algorithm (PPGAA) for baseline wandering. They compared it with several other methods, including independent component analysis (ICA), fast ICA (FASTICA), the Kalman filter, a moving average filter, and a cubic spline algorithm. They added synthetic baseline noise to ECG signals for testing purposes. They found that PPGAA achieved lower mean error values than the other approaches, except ICA, which yielded the best results. Similarly, Ahmed et al. (2023) proposed InvBase, an inverse filtering method for baseline wandering in electroencephalographic (EEG) signals. They evaluated its performance using classification results from a multilayer perceptron in an emotion classification task, demonstrating its superiority over baseline power subtraction and unfiltered signals. For high-frequency noise removal in ECG signals, Krishnamurthy et al. (2015) compared Chebyshev, Butterworth, Hamming, Hanning, Bartlett, and Kaiser filters. They concluded that finite impulse response filters were more effective than infinite impulse response filters, with the Kaiser filter performing better than the others. Additionally, Pandey and Giri (2016) introduced a combined approach that used moving average filtering for noise removal and polynomial curve fitting for signal smoothing. Their method effectively reduced high-frequency noise and was noted for its low computational cost.

Effective analysis of ECG signals requires careful consideration of which parts of the waveform are more relevant, as different cardiac conditions affect various aspects of the heart electrical activity. Analyzing the entire ECG signal or focusing on specific parts can provide valuable insights depending on the studied condition. For example, in arrhythmias such as atrial fibrillation, examining the entire ECG waveform, including the P-wave, QRS complex, and T-wave, is critical for detecting irregular heartbeats and understanding the overall electrical activity of the heart. However, in other conditions, focusing on particular segments of the ECG is more beneficial. For instance, in the case of TWA, it is fundamental to analyze the T-wave, as this is the part of the signal that is specifically affected (Armoundas et al., 2002). Similarly, focusing on the ST segment in diseases like Brugada syndrome is essential, where abnormal elevations may indicate a risk for sudden cardiac events (Antzelevitch, 2006). Given the need for targeted analysis in such conditions, numerous algorithms have been developed to accurately segment the ECG waveform into its different parts. A review of nine automatic ECG wave segmentation algorithms was conducted in Beraza and Romero (2017), where all the algorithms were tested on the same dataset. The review concluded that the best-performing algorithm varied depending on the analyzed ECG segment. For QRS segmentation, the discrete wavelet transform provided the highest sensitivity, whereas algorithms based on the Multi-scale Morphological Derivative and the Phasor transform performed best for segmenting the P-wave and T-wave, respectively. The authors also concluded that Hidden Markov Models (HMM) were the most effective approach for segmenting all waves.

Additionally, several studies have focused on segmenting ECG waves through ML. In Malali et al. (2020), the authors proposed a Convolutional Long Short-Term Memory neural network to segment ECG waves, demonstrating superior performance compared to HMM, particularly for segmenting the P-wave and QRS complex. In Joung et al. (2024), a U-Net was used to localize and segment different waveforms in ECG signals, achieving nearly perfect results for the segmentation of QRS complexes and T-waves. Explicitlyy focusing on T-waves, Sanchez-Carballo et al. (2023) presented the first segmentation method tailored to ECGI data, leveraging the synchronized cardiac activity of the heart. This method takes advantage of the fact that all the signals come from the same patient, allowing for fast and efficient segmentation of the T-wave. Given that this study utilizes this specific data type, we have employed the T-wave segmentation algorithm introduced in it, whose superior performance was quantitatively demonstrated in Sanchez-Carballo et al. (2024).

Concerning the TWA estimation process itself, several recent studies have proposed different approaches for estimating TWA. While these works share a similar goal with our research, i.e., to improve the detection and understanding of TWA through newly developed estimation techniques, they address the challenge of identifying alternans in ECG signals differently. In Ullah et al. (2020), the authors introduced a generalized detection theoretic framework (GDTF) for TWA estimation, which involves preprocessing, data selection, and TWA analysis. The main strengths of this method are that it relies on statistical and probabilistic characteristics of ECG signals, making it more interpretable than many ML techniques, and it provides a clear decision on the presence or absence of TWA. However, like other conventional methods, this approach does not efficiently handle ECGI data, as it cannot effectively integrate this imaging modality temporal and spatial information. In Ullah et al. (2022), the authors presented a piecewise generalized linear modeling (PGLM) approach that does not assume signal stationarity, unlike conventional methods. Instead, it uses first-order piecewise polynomials with cubic spline interpolation, followed by coefficient evaluation via the least squares method. This technique outperforms conventional TWA estimation methods, particularly in noisy ECG signals. However, similar to the GDTF, the PGLM is not suited for ECGI data for the same reasons and does not decide on the presence or absence of TWA, offering only the alternans signal. In Pascual-Sánchez et al. (2024), the authors proposed using K-nearest-neighbor and random forest algorithms to detect TWA, with ML models outperforming the traditional spectral method. Despite these positive results, this approach would not be well-suited for ECGI data, as the ML algorithms employed were designed to learn general patterns rather than subject-specific ones. Our objective, by contrast, is to identify subject-specific features that indicate the presence of TWA, leveraging the greater spatial detail provided by ECGI, which offers a higher number of ECG signals for each subject than conventional ECG studies.

Additionally, due to the increasing interest in using interpretable ML models, recent research has applied SHAP to analyze ML models that process ECG signals, demonstrating its utility in elucidating how different features within the ECG signal impact model predictions. In Rashed-Al-Mahfuz et al. (2021), the authors developed a deep-learning algorithm for cardiac arrhythmia classification. They utilized Shapley values to identify the frequency components in ECG signals that influenced the classifier decisions, thus elucidating the deep learning decision-making process. In Megersa-Ayano et al. (2023), a systematic literature review emphasized that incorporating interpretability algorithms like SHAP into ML models is crucial, particularly for health-related problems where decisions can significantly impact patients’ health. The review concluded that ECG signal classification with interpretable ML models should align with physician reasoning in diagnosing cardiac diseases. Additionally, Anand et al. (2022) demonstrated that the ECG segments identified by SHAP as influential in diagnosing heart diseases aligned with the segments cardiologists focused on, underscoring the practical relevance of SHAP insights in clinical settings. These studies exemplify the potential of SHAP in addressing health-related classification problems with ML algorithms, which has motivated us to develop a customized version of SHAP to understand our algorithm decision-making process better.

After reviewing the literature, we identified numerous TWA analysis studies employing traditional and newly developed estimation methods. However, none of the methods was specifically targeted to ECGI data, which comprises multiple ECG signals rather than a limited set, making existing methods less effective in this context. Leveraging the spatial insights ECGI provides to localize epicardial regions where arrhythmias originate is crucial for advancing non-invasive clinical procedures. This gap in the literature motivated us to develop a subject-specific, interpretable, manifold learning-based approach tailored to ECGI. Our method not only detects TWA in individuals but also identifies the epicardial regions where alternans occur.

## 3. Methods

This section outlines the methodology used in this work. First, the database is introduced. Second, the mathematical foundations of ECGI are explained. Third, the processing stages applied to all ECG signals are described. Fourth, the proposed TWA estimation method is detailed, including the MnL technique for reducing input data dimensions, the community detection algorithm, and the hypothesis test based on Bootstrap resampling. Finally, the customized algorithm designed to enhance the explainability of our approach is discussed.

**Fig. 1.**
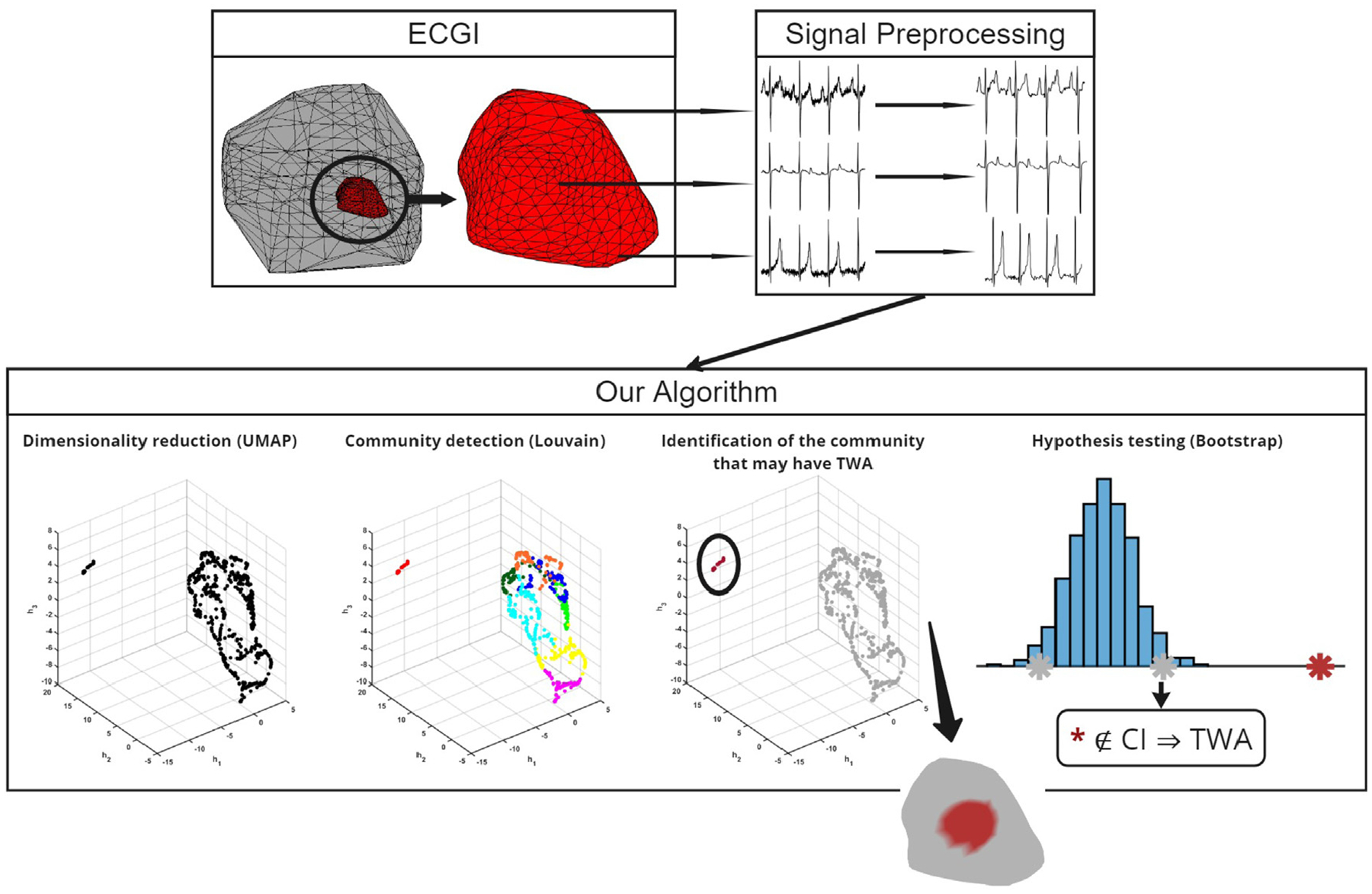
General schema describing the workflow pipeline. It begins with a discretized mesh containing N mesh points. Next, the signals are preprocessed and prepared for input into our algorithm. Finally, the algorithm involves reducing the dimensions of the original data with UMAP, detecting the different communities with the Louvain algorithm, identifying the community that may have TWA, and evaluating a hypothesis test to determine if TWA exists.

Fig. 1 summarizes the work, focusing on the novel MnL-based TWA detection algorithm. ECGI epicardial data was obtained by estimating the heart electrical activity from body surface potential measurements, combined with the subject anatomical information. This resulted in constructing an epicardial mesh consisting of multiple nodes associated with an ECG signal, thus enhancing spatial resolution. All epicardial signals underwent a uniform preprocessing step in which they were detrended and low-pass filtered. Afterward, even and odd T-waves were segmented, and T-wave templates for each were generated at each mesh point, serving as input for the proposed method. The first step of the algorithm involves reducing the dimensionality of the original data using UMAP, which preserves both local and global data structures. In the second step, the algorithm detects communities in the embedded space using the Louvain algorithm, which accounts for the topology of the lower-dimensional UMAP graph during community detection. Finally, in the third step, the community exhibiting the most TWA is identified within the epicardial mesh and compared with other communities using Bootstrap resampling to reinforce the statistical reliability of the decision regarding the presence or absence of alternans.

### 3.1. Database description

The ECGI database used in this work contained recordings from two control subjects, eight patients with LQTS and seven patients with ICM. All the data were acquired at the Cardiac Bioelectricity and Arrhythmia Center, Yoram Rudy Lab at Washington University in St. Louis, as part of previous studies (Cuculich et al., 2011; Vijayakumar et al., 2014). Torso measurements were extracted using body surface potential mapping, and epicardial potentials were estimated by solving the inverse problem of electrocardiography (Ramanathan et al., 2004). Additionally, four new patients were generated to create cases with known solutions, allowing us to assess better the quality of the method employed. Two of these patients were synthetically created by repeating the first beat of each ECG signal from the two control subjects twenty times and including synthetic alternans in a specific epicardial area. The other two patients were created by adding synthetic alternans to a specific epicardial area of the control subjects’ epicardial meshes without altering them. Additional details on the generation of these patients are included in Sanchez-Carballo et al. (2024). The data did not contain further information, and it only included the subject’s ECGI measurements. All subjects signed the corresponding informed consent, and the protocols were reviewed and approved by the Human Research Protection Office at Washington University in St. Louis. The data were acquired under NIH–NHLBI grant numbers R01-HL033343 and R01-HL-049054, awarded to Prof. Yoram Rudy. Table 1 summarizes the characteristics of the data used in this work. For each individual, the number of nodes in the epicardial mesh, the duration of the ECG signals in seconds, the T-waves duration in seconds, and the number of beats in the ECG signals are included.

### 3.2. Electrocardiographic imaging

As previously explained, ECGI is a non-invasive imaging technique that maps the electrical activity of the heart by integrating torso potential measurements with anatomical information, usually obtained from CT or MRI scans. It creates a detailed representation of the epicardial surface, allowing for the analysis of complex cardiac electrical patterns (Rudy and Burnes, 1999). Let $S^{2}$ be a two-dimensional continuous surface located in a three-dimensional space, representing the epicardium or the torso. Let $r_{S^{2}}$ be the set of points in surface $S^{2}$, defined as follows,

(1) $$r_{S^{2}}\equiv \left\{r\in S^{2},S^{2}\in R^{3}\right\}$$

where r is the position vector of any point in the three-dimensional space. Now, let the potential fields that change with time be denoted as $v_{S^{2}}=v\left(r_{S^{2}},t\right)$. The continuous surface $S^{2}$ should be discretized to define a geometrical mesh. In this context, each node i that belongs to the mesh can be defined as follows,

(2) $$s_{i}=r_{S^{2}}\cdot \delta \left(r-r_{i}\right)$$

where δ(⋅) is the Dirac delta function in the spatial domain, and $\left\{s_{i},i=\right.1,2,\ldots ,N\}$ is the discrete set of N nodes that belong to surface $S^{2}$. Accordingly, the discretized mesh can be described as follows,

(3) $$r_{S^{2}}^{N}=r_{S^{2}}\sum_{i=1}^{N}\delta \left(r-r_{i}\right)$$

The ECGI data used in this study was obtained by recording torso potentials using a 256 electrodes vest. While wearing the vest, each subject underwent a thoracic CT scan, allowing for the acquisition of patient-specific heart-torso geometry. By combining this anatomical information with body surface potentials and applying custom-developed algorithms, cardiac electrical potentials were non-invasively reconstructed as follows,

(4) $$V^{E}=M^{-1}\cdot V^{B}$$

where $V^{E}$ and $V^{B}$ represent the epicardial and body surface potentials, respectively, and matrix M contains the geometric relationship between the heart and the torso. This equation represents the inverse problem of electrocardiography.

### 3.3. Signal preprocessing

Once the epicardial potentials were estimated, filtering them out was necessary. Specifically, two different filters were applied to all ECG signals. First, a spline detrending filter was applied. This filter estimated the signal trend by fitting a smooth curve to windowed averages of the signal and then subtracting this trend from the original signal to produce a detrended version. To implement the filter, a moving average was calculated over 50% overlapping windows, with the window length set to one heartbeat. After obtaining the moving averages, a smoothing spline was fitted using cubic spline approximation. This method smoothed the data by interpolating between the averaged points while minimizing curvature, allowing the spline to capture more complex, slowly varying trends rather than a simple linear trend. The spline was evaluated at each time point of the original signal, providing an estimated trend across the entire signal. Finally, the estimated trend was subtracted from the original signal, effectively removing slowly varying components and isolating the oscillatory or high-frequency elements of the signal. After detrending the signals, a low-pass filter was applied to remove high-frequency noise while preserving relevant signal components. A Butterworth finite impulse response filter with an order of 150 and a cutoff frequency of 30 Hz was designed. The filter coefficients were obtained by normalizing the cutoff frequency relative to half the sampling frequency, following the Nyquist criterion, ensuring attenuation of frequencies above 30 Hz. To prevent phase distortion, zero-phase filtering was used, where the signal was filtered forward and backward. This process preserved the timing characteristics of the ECG signal. The filter order 150 provided a sharp transition between the passband and stopband, ensuring sufficient attenuation of frequencies above 30 Hz while preserving the integrity of lower-frequency components, such as TWA, which typically occur below this threshold. This step improved signal quality by eliminating unwanted high-frequency noise without introducing phase distortion, thus facilitating the preservation of relevant physiological features. The signals were analyzed before and after filtering to ensure that no physiological information related to the T-waves was erased or modified. Consistently with the previous notation, the potential fields were calculated as follows,

(5) $$V^{\prime }=f_{BW}\left(f_{OB}(V)\right)$$

where $V^{\prime }$ represents the filtered potentials and $f_{BW}$ and $f_{OB}$ are the detrending and out-band noise removal operators, respectively.

After filtering the signals, T-waves were segmented using the Single Reference Segmentation method (Sanchez-Carballo et al., 2023). This method is specific to ECGI data, leveraging the synchronous activity across the cardiac muscle. For each individual and mesh node i, even and odd T-wave templates, denoted as ${\bar{t}}_{e}^{i}$ and ${\bar{t}}_{o}^{i}$, respectively, were generated by averaging its even and odd T-waves, respectively. For more information about the preprocessing steps, refer to the software publicly available in https://github.com/estelasc/TWA-analysistoolbox.


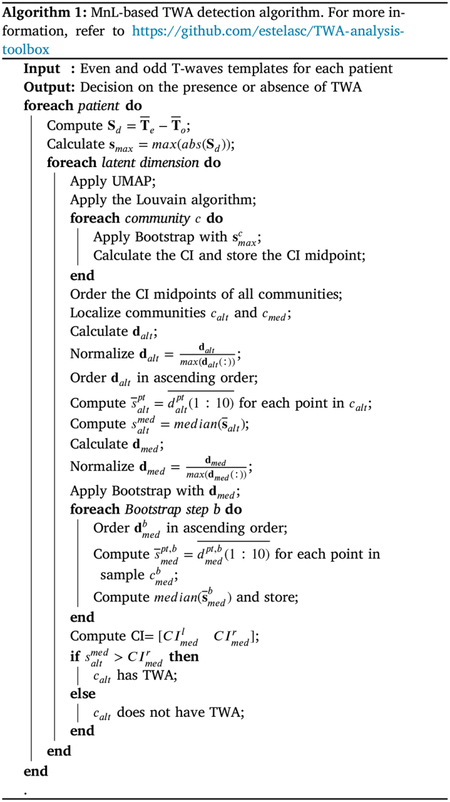


### 3.4. T-wave alternans detection algorithm

In this work, we propose a novel MnL-based TWA estimation method, which is the first designed explicitly for ECGI data. To detect TWA in specific regions of the cardiac muscle, our algorithm uses UMAP to reduce data dimensions, the Louvain algorithm to identify communities, including the one with the most TWA, and Bootstrap resampling to test whether the identified community exhibits TWA. This subsection explains the different methods used by our algorithm. Again, refer to https://github.com/estelasc/TWA-analysis-toolbox, where the entire algorithm is implemented.

The set of N even and N odd templates was the input of the proposed algorithm, which is described in Algorithm 1. The objective of Algorithm 1 is to analyze TWA within a low-dimensional space that captures the essential features of each ECG signal from the same individual, along with the specific geometrical characteristics unique to that subject. Reducing dimensions simplifies the analysis, and in this work, we rely on UMAP to do so, as this algorithm excels at maintaining the original data structure in the latent space. UMAP is a non-linear dimensionality reduction algorithm that projects the data into a lower-dimensional space, preserving both their local and global structures. The method achieves this by generating a high-dimensional graph representation of the data and optimizing a low-dimensional graph to be as structurally similar as possible to the higher-dimensional counterpart. Mathematically, UMAP uses non-normalized exponential probabilities to characterize the similarity between two points $x_{i}$ and $x_{j}$ in the original space, i.e.,

(6) $$p_{ij}=p_{i|j}+p_{j|i}-p_{i|j}p_{j|i}$$

where

(7) $$p_{j|i}=e^{-\frac{\left\|x_{i}-x_{j}\right\|-\rho_{i}}{\sigma_{i}}}$$

where $\left\|x_{i}-x_{j}\right\|$ is the Euclidean distance, $\rho_{i}$ represents the distance from the ith data point to its nearest neighbor, and $\sigma_{i}$ is a scaling parameter specific to each data point i. This formula combines the conditional probabilities to adjust for their mutual overlap, ensuring that the result is symmetric and represents the similarity between points i and j. This symmetrization process has two main advantages. The first one is that it provides a balanced representation of similarities, which is important for accurately capturing the underlying structure of the data. The second one is that it helps avoid biases that might arise if only one of the conditional probabilities were used, ensuring that the relationship between pairs of points is mutual and consistent.

In the embedded space, the similarity between points $y_{i}$ and $y_{j}$ is modeled as

(8) $$q_{ij}={\left(1+a\cdot {\left\|y_{i}-y_{j}\right\|}^{2b}\right)}^{-1}$$

where parameter a corresponds to the scaling parameter that controls the influence of the distance term, and parameter b refers to the shape parameter that controls the steepness of the decay in the probability as distance increases. By definition, these parameters must be positive, and by default, a≈1.93 and b≈0.79 are used. However, it has been demonstrated that setting a=b=1 has no qualitative effect on the results (Ghojogh et al., 2023). UMAP determines these parameters through non-linear least-square fitting to the piecewise function while setting a minimum distance between points, denoted as $d_{0}$. This is achieved as follows,

(9) $$q_{ij}\approx \left\{\begin{array}{ll} 1 & \text{for}\;\left\|y_{i}-y_{j}\right\|\leq d_{0}\\ e^{-\left\|y_{i}-y_{j}\right\|-d_{0}} & \text{for}\;\left\|y_{i}-y_{j}\right\|>d_{0} \end{array}\right.$$

This probability function helps to ensure that points close in the lower-dimensional space have higher probabilities of being neighbors, while points far apart have lower probabilities. It ensures that the embedding visually represents the original high-dimensional relationships.

The UMAP cost function is the binary cross entropy (BCE) between the joint probability distribution $p_{ij}$ in the high-dimensional space and the joint probability distribution $q_{ij}$ in the low-dimensional space, i.e.,

(10) $$C=\sum_{i}\sum_{j}p_{ij}\;\text{log}\;\left(\frac{p_{ij}}{q_{ij}}\right)+\left(1-p_{ij}\right)\;\text{log}\;\left(\frac{1-p_{ij}}{1-q_{ij}}\right)$$

which is minimized using the stochastic gradient descent algorithm. The cost function in UMAP is designed to minimize the difference between the high-dimensional probability distribution $p_{ij}$, which represents the pairwise similarities between points in the high-dimensional space, and the low-dimensional probability distribution $q_{ij}$, which represents the pairwise similarities in the lower-dimensional space. Specifically, the BCE loss function is used to align these distributions. The first term of the BCE function aims to attract the embeddings of neighboring points toward each other and only takes effect if $x_{i}$ is a neighbor of $x_{j}$ or vice versa, or if both points are neighbors. On the other hand, the second term of the function repels the embeddings of non-neighboring points, pushing them away from each other. By minimizing the BCE loss, UMAP aims to ensure that the local and global structure of the data is preserved as accurately as possible during dimensionality reduction.

In the UMAP notation, the original data matrix X for each individual can be defined as follows,

(11) $$X={\left[x_{1},x_{2},\ldots ,x_{N}\right]}^{T}$$

On the other hand, in our specific case, the set of even and odd T-wave templates for each subject can be expressed as follows,

(12) $${\bar{T}}_{e}={\left[{\bar{t}}_{e}^{1},{\bar{t}}_{e}^{2},\ldots ,{\bar{t}}_{e}^{N}\right]}^{T}\;\text{and}\;{\bar{T}}_{o}={\left[{\bar{t}}_{o}^{1},{\bar{t}}_{o}^{2},\ldots ,{\bar{t}}_{o}^{N}\right]}^{T}$$

To apply UMAP, we compute the difference signals, also known as alternans signals, denoted as $S_{d}$, which were calculated as follows,

(13) $$S_{d}={\bar{T}}_{e}-{\bar{T}}_{o}$$

Therefore, the original data represented by matrix X in the UMAP notation corresponds to matrix $S_{d}$ in this more specific notation, which contains the information of the alternans signals.

As previously stated, it was hypothesized that ECG signals with TWA would be located close to each other in the embedded space. By clustering these lower-dimensional data points, an analysis can be performed on the areas of the cardiac geometry that exhibit TWA. For this purpose, our algorithm uses the UMAP-optimized graph for community detection, specifically employing the Louvain algorithm, characterized by its efficiency and robustness in creating communities. Explicitly, this community detection method extracts non-overlapping communities by maximizing the graph modularity (GM), which is calculated as follows,

(14) $$GM=\frac{1}{2\cdot m}\cdot \sum_{ij}\left(\left[A_{ij}-\frac{k_{i}\cdot k_{j}}{2\cdot m}\right]\cdot \delta \left(c_{i},c_{j}\right)\right)$$

where m is the number of edges in the graph, $A_{ij}$ is a binary variable whose value is 1 when nodes i and j are connected by an edge and 0 when they are not connected, $k_{i}$ is the number of connections of node i, and $\delta \left(c_{i},c_{j}\right)$ is another binary variable that equals one if nodes i and j belong to the same community and 0 if they do not, forcing the GM to be null if nodes i and j belong to different communities. The GM is a metric that measures the density of connections between different communities and functions as the cost function to maximize in the Louvain algorithm. The Louvain algorithm initially assigns each node its own community. Then, every node i is considered for assignment to its neighboring node j, and the GM is recalculated. This occurs in the local optimization phase, where the change in modularity from moving node i from its original community to another community c, denoted as ΔGM, is computed as follows,

(15) $$\Delta GM=\left[\frac{\sum_{in,c}+k_{i}^{in}}{2\cdot m}-{\left(\frac{\sum_{tot,c}+k_{i}}{2\cdot m}\right)}^{2}\right]-\left[\frac{\sum_{in,c}}{2\cdot m}-{\left(\frac{\sum_{tot,c}}{2\cdot m}\right)}^{2}\right]$$

where $\sum_{in,c}$ is the sum of the weights of edges within community $c,k_{i}^{in}$ is the sum of the weights of edges between node i and other nodes in community $c,\Sigma_{\text{tot},c}$ is the sum of the degrees of all nodes in community c, and $k_{i}$ is the total weight of edges connected to node i. The algorithm iterates over all nodes, moving them among communities to maximize the GM until no further improvement can be made. Once the local optimization phase is completed, the aggregation phase starts, where the Louvain algorithm groups the nodes into communities and assigns each community to a new, single node, creating a new network where each node represents a community from the previous phase. The edge weights between the new nodes are equal to the sum of the edge weights between the corresponding communities in the original network. These two phases of the Louvain algorithm are iteratively repeated until the GM cannot be increased further, producing the final community structure.

**Fig. 2.**
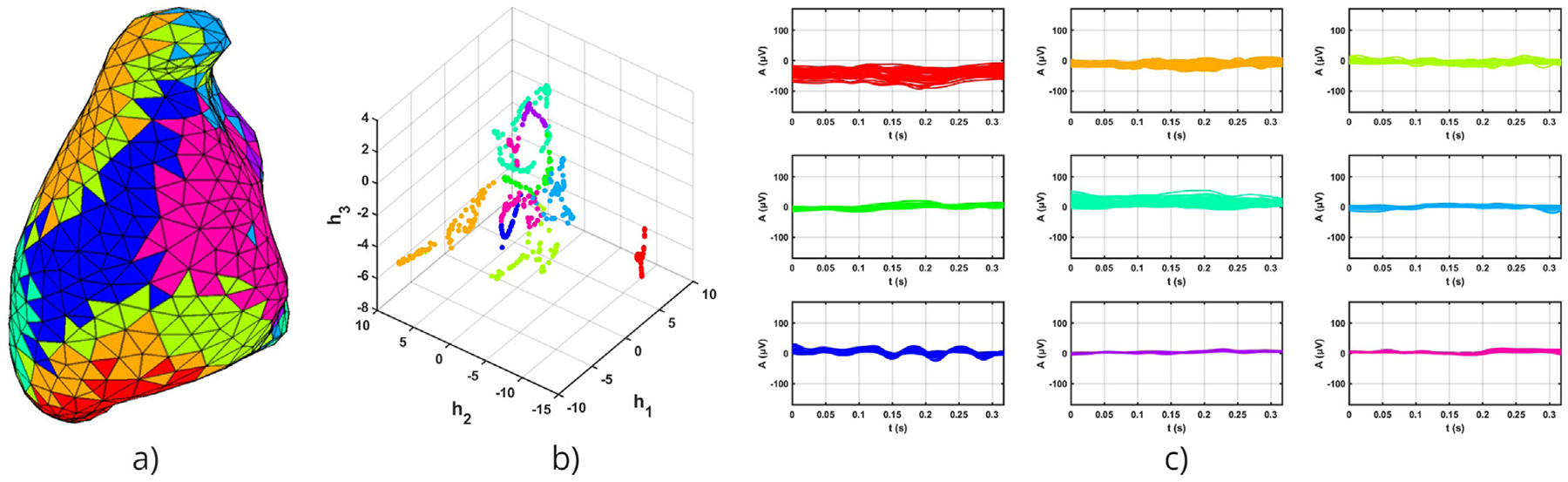
Visual representation of the epicardial mesh (a), the lower-dimensional space generated by UMAP (b), and the alternans signals associated with data points in the latent space, after the application of the Louvain algorithm (c). The data points are grouped into different communities, localized in the epicardial mesh and the embedded space. Signals are classified according to their respective communities, with each community depicted in a different color.

Fig. 2 illustrates the division of signals into multiple communities. Our algorithm identifies the community with the highest TWA and treats the remaining communities collectively. However, there may be more than two communities in the figure, each represented by a different color. Each community is associated with specific nodes in the mesh (a), a location in the latent space (b), and corresponding input signals (c), where each signal corresponds to a particular node in the mesh and a data point in the embedded space. Panel (c) demonstrates how the data points grouped into the same community by the Louvain algorithm correspond to alternans signals that are very similar to each other and distinct from signals in different communities.

Once the existing communities have been identified, our algorithm focuses on localizing the community that exhibits TWA. In this context, it is assumed that the Louvain algorithm has grouped all the data points with TWA together if any of them exhibit it. Bootstrap resampling is employed to decide which community is the candidate to contain the signals with TWA and whether they show it. Specifically, Bootstrap is performed for each community c using the set of maximum values of the difference signals that belong to c, i.e., $s_{\text{max}}^{c}$, as the input for the Bootstrapping algorithm. For each community, g values are taken from $s_{\text{max}}^{c}$ with replacement, where g is the number of nodes in community c, and then the mean of those values is computed. This process is repeated 500 times, and the 95% CI is calculated using Bonferroni correction. Each community CI midpoint is also calculated. Our algorithm focuses on the community with the highest CI midpoint, denoted as $c_{\text{alt}}$, which is hypothesized to exhibit TWA, and on the community with median CI midpoint, denoted as $c_{\text{med}}$. The hypothesis to study is the following,

(16) $$\left\{\begin{array}{l} H_{0}:c_{\text{alt}}\;\text{is not different enough from the other communities}\;(\text{no TWA})\\ H_{1}:c_{\text{alt}}\;\text{is different enough from the other communities}\;(\text{TWA}) \end{array}\right.$$

To analyze the hypothesis in Eq. (16), the Euclidean distances between the points belonging to $c_{\text{alt}}$ and the rest of the points in the lower-dimensional space are calculated, denoted as $d_{\text{alt}}$, then normalized and ordered. After that, the median of the mean of the ten smallest distances for each point pt in $c_{\text{alt}}$ is computed, i.e., ${\overset{‾}{s}}_{\text{alt}}^{\text{med}}$. A similar process is followed with community $c_{\text{med}}$. The Euclidean distances between the points belonging to $c_{\text{med}}$ and the rest of the points in the lower-dimensional space are calculated, obtaining $d_{\text{med}}$, and normalized. Still, then they undergo a Bootstrap resampling process. Specifically, in each Bootstrap step, h distances for each point belonging to $c_{\text{med}}$ are taken with replacement, where h is the number of points that do not belong to $c_{\text{med}}$, and then the median of the mean of the ten smallest distances for each point in $c_{\text{med}}$ is computed. This process is repeated 500 times, and the 95% CI, i.e., $\left[CI_{\text{med}\;}^{l}CI_{\text{med}}^{r}\right]$, is calculated. The presence or absence of TWA is determined as follows,

(17) $$H_{0}=\left\{\begin{array}{lll} \text{false} & \text{if} & s_{\text{alt}}^{\text{med}}>CI_{\text{med}}^{r}\\ \text{true} & \text{if} & s_{\text{alt}}^{\text{med}}\leq CI_{\text{med}}^{r} \end{array}\right.$$

In this manner, the null hypothesis $H_{0}$ described in Eq. (16) is proven to be either true or false, and consequently, the alternative hypothesis is proven to be the contrary.

### 3.5. Customized explainability method

To enhance the interpretability of our algorithm, we developed a customized version of SHAP, which assigns a contribution value to each feature, specifically to each latent space variable in our model, indicating its influence on the output. This method ensures consistency in feature importance, enabling clear explanations of the final decisions made by ML models. SHAP provides mathematically grounded attributions for each feature, increasing transparency and trust in the model, which is especially valuable in applications where understanding the reasoning behind predictions and decision-making processes is critical. In the original work (Shapley, 1953), Shapley values were defined as

(18) $$\phi_{j}(f)=\sum_{S\subseteq \{1,\ldots ,p\}\setminus \{j\}}\frac{|S|!(p-|S|-1)!}{p!}(f(S\cup \{j\})-f(S))$$

where $\phi_{j}(f)$ is the Shapley value of the jth feature evaluated with the measure f(⋅),S is a subset of features used in our model, p is the number of features, and f(⋅) is the function we want to evaluate.

However, this classical and well-established method exhibits two main drawbacks in our application. First, its computational cost grows exponentially with the number of features, and since we are working with time series, the original number of samples often exceeds 500. Second, the way Shapley values are computed disrupts the temporal coherence of the signal, making it unsuitable for time series data. To address these limitations, we propose using a customized version of the original SHAP algorithm, known as WindowSHAP (Nayebi et al., 2023), which works with temporal windows rather than individual samples, preserving temporal coherence across windows. In this work, we combine this method with a Monte Carlo-based algorithm that enables the computation of Shapley values within a reasonable time (Štrumbelj and Kononenko, 2014). Thus, the interpretability algorithm used in this work can be formalized as follows,

(19) $$\phi_{w}(f)=\frac{1}{M}\sum_{m=1}^{M}\left(f\left(x_{+w}^{m}\right)-f\left(x_{-w}^{m}\right)\right)$$

where $x_{+w}^{m}$ represents an instance of our input signal in which a random number of temporal windows are replaced by windows from a random signal from the set, except for the value of the wth window, and $f\left(x_{-w}^{m}\right)$ is a copy of the previous variable in which the value of the wth window is also replaced. From now on, we will refer to this explainability algorithm as MC-WindowSHAP. In this work, we applied MC-WindowSHAP to signals in $c_{\text{alt}}$ to understand why they were separated from the remaining signals in the embedded spaces. However, to ensure comprehensive analysis and accurate feature attribution, we included all signals, not just those with TWA. This inclusion allowed us to perform the necessary substitutions and comparisons across the entire dataset, thus enhancing the robustness of the feature importance insights derived from MC-WindowSHAP. For more information about implementing this customized explainability method, refer to https://github.com/estelasc/TWA-analysis-toolbox.

## 4. Experiments and results

In this section, we present the experiments conducted during the development of this work, focusing on optimizing and evaluating the performance of the proposed subject-specific algorithm. We begin by examining the effect of the preprocessing steps on the different ECG signals. Next, we describe the process of selecting the most appropriate number of dimensions for reducing the input data. To achieve this, UMAP was applied independently to each subject, and the optimal number of dimensions was determined by collectively analyzing and comparing the results across all subjects. Following this, we present the algorithm results, emphasizing its interpretability on a subject-specific basis and highlighting the results supporting the Bootstrap-based TWA decision process, which was also evaluated subject-specific to ensure personalized analysis. Finally, we applied a customized version of the well-known SHAP interpretability algorithm, offering an explanation of how our algorithm determines whether a patient exhibits TWA.

### 4.1. Filtering effect

**Fig. 3.**
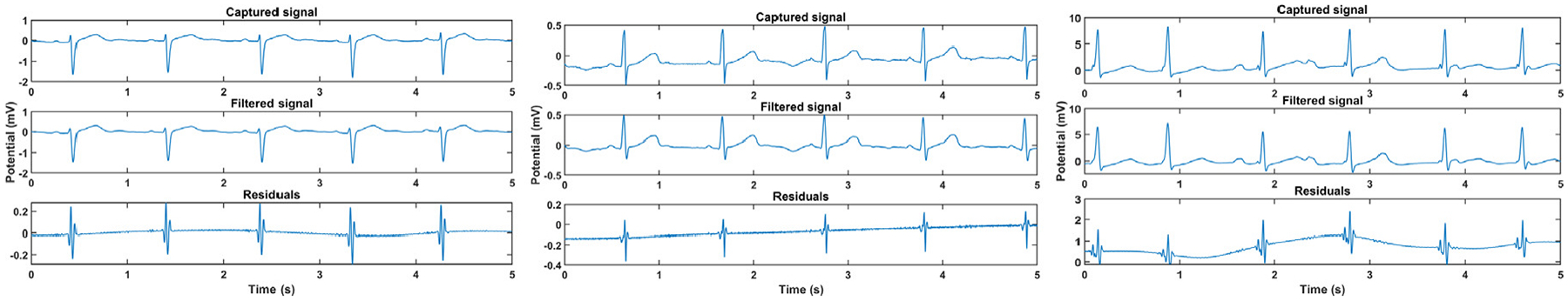
Original ECG signals (top), filtered signals (middle), and residuals (bottom) for one control subject (left), one patient with LQTS (middle), and one patient with ICM (right).

When analyzing TWA, special attention should be given to the preprocessing steps, as noise could be misinterpreted as pathological features related to TWA. In this study, the preprocessing filters were carefully designed to remove as much noise as possible while preserving the integrity of the T-wave, which is the most relevant ECG segment for TWA analysis. Special care was taken to ensure no crucial T-wave information was lost. To demonstrate the effectiveness of these preprocessing steps, Fig. 3 illustrates the impact of filtering on different ECG signals. The figure shows an ECG signal before filtering (top), after filtering (middle), and the residuals (bottom) for a control subject (left), a patient with LQTS (middle), and a patient with ICM (right). The difference between the original and filtered signals is displayed in the lower panels. These plots clearly show that the T-wave remained unaffected by the filtering process, confirming that no crucial information related to the T-wave was lost. Notably, the only physiological information removed by the filters was related to the QRS complex, as indicated by the residuals, confirming that only noise and irrelevant ECG components were eliminated. This ensures that the preprocessing step effectively cleanses the signals without distorting the T-wave.

### 4.2. Parameter tuning for the algorithm

Before applying the novel MnL-based TWA detection method, an experiment was conducted to determine the most appropriate number of dimensions for UMAP to reduce the input data. To achieve this, Algorithm 1 was applied to all patients up to the step where the Euclidean distance between community $c_{\text{alt}}$ and the rest of the points in the embedded space was calculated and normalized. Specifically, for each patient, each ECG signal was projected into three-, six-, nine-, twelve-, fifteen-, eighteen-, and twenty-one-dimensional spaces. The Louvain algorithm was applied in each case, distances were calculated and normalized, and the median of the mean of the ten smallest distances for each projection was computed. These distances are shown in Table 2, where subject identifiers are listed in the first column, and the dimensions of the latent spaces are provided in the first row. The last row displays the mean normalized distance for each dimension, excluding control subjects C1 and C2, as they are healthy, and synthetic patients with added synthetic TWA, as localizing TWA was straightforward in these cases. Bolded and italicized values in Table 2 represent the highest and second-highest values for each patient, respectively. We hypothesized that $c_{\text{alt}}$ contained the signals where TWA was present, and reducing the data to eighteen dimensions generally produced a more different cluster of those signals, as it yielded the highest or second-highest value in thirteen out of seventeen cases. This was followed by dimensions fifteen, twenty-one, and twelve, as shown in the last row of Table 2.

**Fig. 4.**
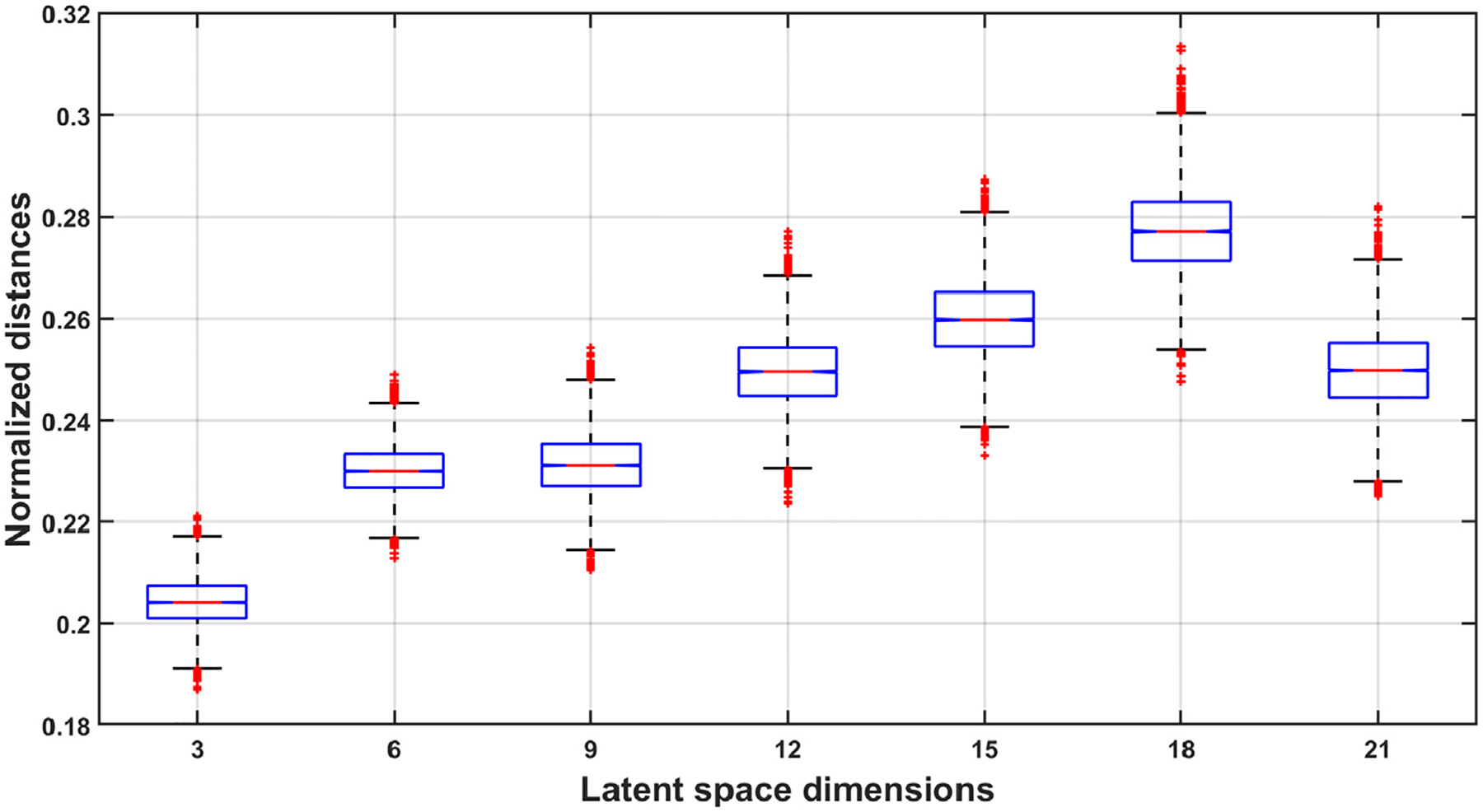
Mean ± standard deviation intervals of the normalized distances for each latent space dimension. Bootstrap is used to derive the metrics.

Fig. 4 was generated by applying Bootstrap resampling to the distances in Table 2, separately for each dimension. Bootstrap resampling provides a more robust estimate of key distributional features, such as quartiles, by mitigating the distortions associated with small sample sizes. This enhances the accuracy and reliability of the results. In this context, Fig. 4 supports the same conclusion as Table 2, i.e., reducing the dimensions to eighteen offered the best separation of the community with alternans from the other communities. Based on these findings, the dimensions of the input data were reduced to eighteen in the remaining experiments. This step can be understood as a free parameter tuning stage, performed to optimize the algorithm performance.

### 4.3. T-wave alternans detection algorithm results

**Fig. 5.**
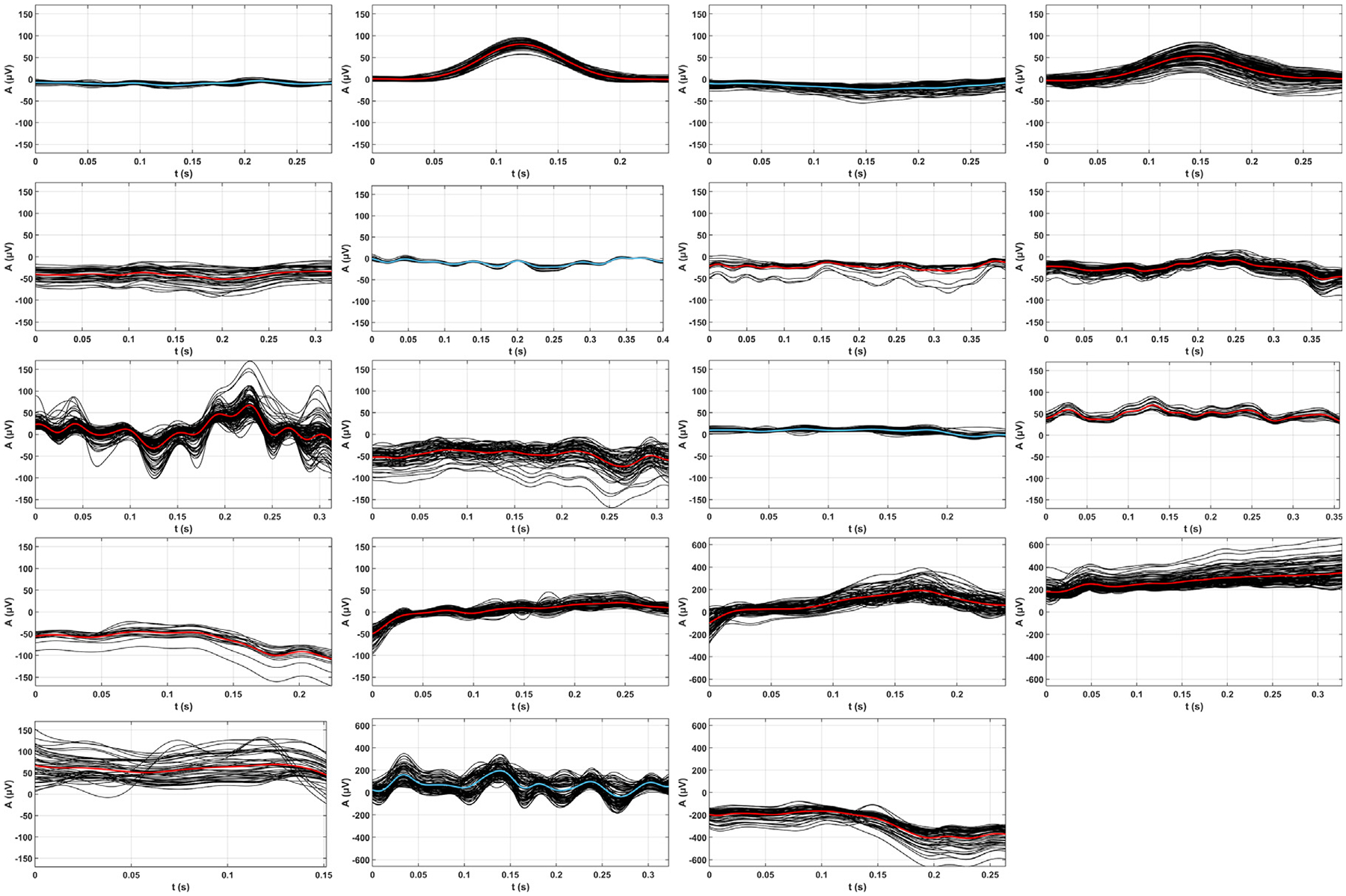
Even–odd T-wave differences in the nodes belonging to the community where they are greater. Results correspond to control subjects 1 and 2, first without being altered and then with synthetic TWA (first row), LQTS Patients 1 to 4 (second row), LQTS Patients 5 to 8 (third row), ICM Patients 1 to 4 (fourth row), and ICM Patients 5 to 7 (fifth row), arranged from left to right.

Once the number of dimensions for UMAP to reduce the original data was selected, Algorithm 1 was applied on a subject-by-subject basis. Table 3 shows which patients were identified by our method as exhibiting TWA. The success rate of the decisions in cases with known outcomes was the highest. TWA was not detected in control subjects C1 and C2. In contrast, synthetic patients with synthetic TWA and control subjects with synthetic TWA were correctly identified as having TWA. Among the patients, TWA was detected in 80% of them. Fig. 5 displays the signals that belonged to $c_{\text{alt}}$ for each patient. Analyzing the signals from the different subjects allowed for a better understanding of the decisions made by the algorithm. For those subjects for whom our algorithm detected no TWA, the mean signal is highlighted in blue in the figure and otherwise in red. In control subjects C1 and C2 (first row, first and third columns), the mean signal is centered at 0μV, indicating no differences between even and odd T-waves. Thus, our algorithm concluded that these subjects did not exhibit TWA. The algorithm also rejected the hypothesis that LQTS Patients 2 and 7 (second row, second column, and third row, third column, respectively) have TWA, as the signals belonging to $c_{\text{alt}}$ in these patients are also centered around 0μV. Regarding ICM Patient 6, it can be seen that the signals in $c_{\text{alt}}$ are not centered at 0μV, but the algorithm decided that this patient did not exhibit TWA. This case will be further analyzed later, but the reason is that all the signals belonging to this patient were similarly noisy, making the signals in $c_{\text{alt}}$ not notably different from those in other communities. In the remaining patients, the $c_{\text{alt}}$ signals are not centered at 0μV. Indeed, two types of TWA behaviors can be observed, i.e., hump-shaped TWA and amplitude-shifted TWA. Examples of the first case include subjects with synthetically added TWA (first row, second and fourth columns), LQTS Patient 4 (second row, fourth column), and ICM Patients 2 and 3 (fourth row, second and third columns, respectively). Examples of the second case are LQTS Patients 1, 3, 6, and 8 (second row, first and third columns, and third row, second and fourth columns, respectively) and ICM Patients 1, 4, and 5 (fourth row, first and fourth columns, and last row, first column). LQTS Patient 5 (third row, first column) and ICM Patient 7 (last row, third column) represent a mixed case between hump-shaped TWA and amplitude-shifted TWA.

**Fig. 6.**
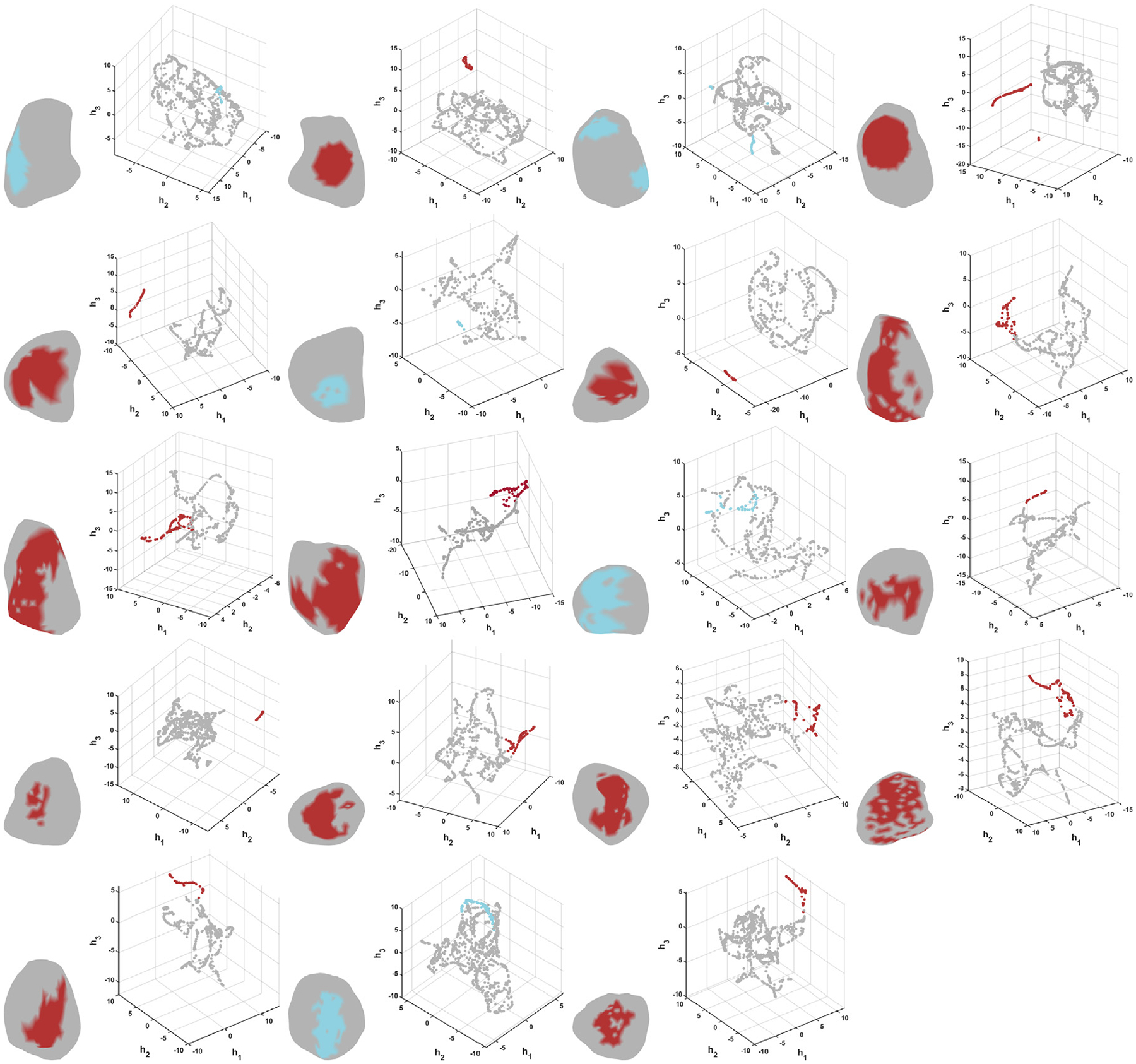
Meshes and latent spaces, with the community showing greater even–odd T-wave differences highlighted either in red (indicating the presence of TWA according to the Bootstrap-based proposed method) or in blue (indicating the absence of TWA according to the Bootstrap-based proposed method). Results correspond to control subjects 1 and 2, first without alterations and then with synthetic TWA (first row), LQTS Patients 1 to 4 (second row), LQTS Patients 5 to 8 (third row), ICM Patients 1 to 4 (fourth row), and ICM Patients 5 to 7 (fifth row), arranged from left to right. For visualization purposes, eighteen-dimensional latent spaces were reduced to three-dimensional spaces.

Fig. 6 illustrates the epicardial meshes and latent spaces generated by UMAP, with community $c_{\text{alt}}$ highlighted in blue if our algorithm detected no TWA or in red otherwise. For the control subjects and the patients in whom TWA was not detected, it can be observed that community $c_{\text{alt}}$ is not separated from the other data points in the latent spaces. Instead, it belongs to the main cluster along with the other communities. However, this is not the case for the patients for whom our algorithm identified TWA. In the latent spaces of synthetic subjects with added TWA, LQTS Patients 1 and 3, and ICM Patient 1, community $c_{\text{alt}}$ form a clear, distinct cluster separated from the main one. In the latent spaces of LQTS Patients 4, 5, 6, and 8, and ICM Patients 2, 3, 4, 5, and 7, the community $c_{\text{alt}}$ cluster is not as separated from the main group as in the other cases. However, it is still differentiated, always located at one border of the main cluster. Note that, for visualization purposes, the eighteen-dimensional spaces generated by UMAP were reduced to three-dimensional representations.

**Fig. 7.**
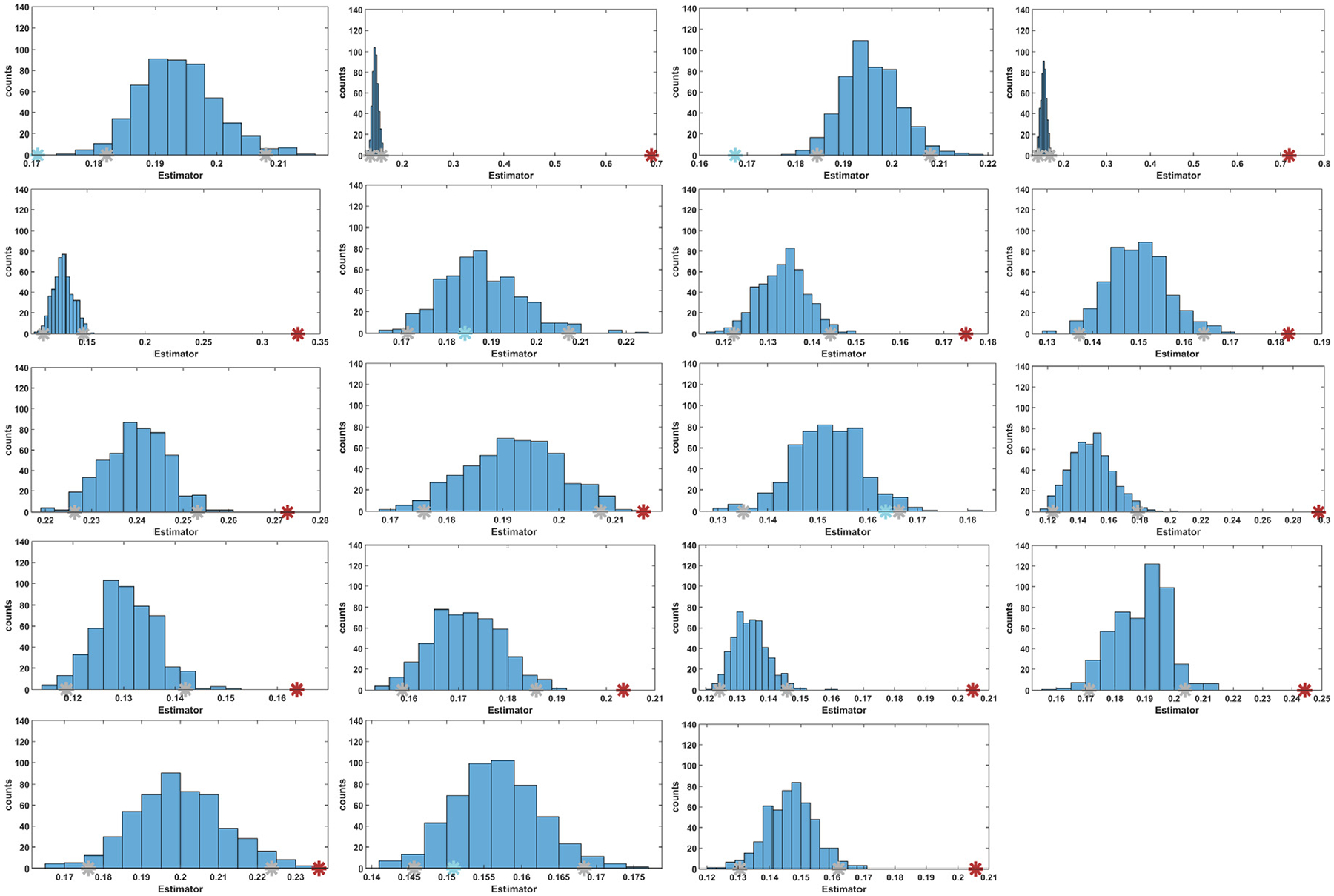
Histograms of the Bootstrap-based proposed method output, with the CI highlighted in gray and the target value highlighted in either red (when the method recognizes TWA) or blue (when the method does not recognize TWA). Results correspond to control subjects 1 and 2, first without alterations and then with synthetic TWA (first row), LQTS Patients 1 to 4 (second row), LQTS Patients 5 to 8 (third row), ICM Patients 1 to 4 (fourth row), and ICM Patients 5 to 7 (fifth row), arranged from left to right.

For the decision-making process, the final part of Algorithm 1 analyzed whether community $c_{\text{alt}}$ exhibited TWA using Bootstrap resampling. For each subject, Fig. 7 displays the distribution obtained through Bootstrap, with the CI shown in gray and the empirical value for $c_{\text{alt}}$ depicted in blue if it is to the left of the right limit of the CI, or in red otherwise. In subjects where TWA was not detected, the empirical value fell within the CI or was even to the left of the left limit of the CI in the case of the control subjects, i.e., in no case did it overlap with the right limit of the CI. In cases where TWA was detected, the empirical value did not overlap with the right limit of the CI either. Specifically, empirical values in these were approximately 0.007 to 0.19 points away from the upper limit of the CI, with a mean of 0.049 and a median of 0.026. They were sufficiently shifted to the right in all cases, being completely outside the distribution in approximately 85% of them.

**Fig. 8.**
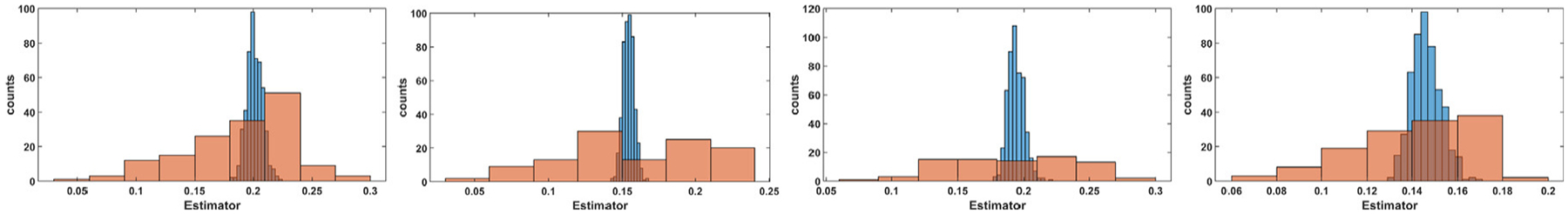
Histograms of the Bootstrap-based proposed method output (blue) and the distribution obtained without using Bootstrap resampling (orange). Results correspond to control subjects 1 and 2, first without alterations and then with synthetic TWA, arranged from left to right.

Fig. 8 shows the data distributions for control subjects C1 (left) and C2 (right), without being altered and with synthetic TWA, as presented in Fig. 7. The blue distributions represent the data obtained using Bootstrap resampling, while the orange distributions represent the data without Bootstrap resampling. The distribution obtained without Bootstrap is clearly wider than the one obtained using the resampling technique. This may indicate that the non-Bootstrap distribution is not adequately capturing the true data variability due to the small sample size. When the number of observations is limited, the resulting distribution becomes sensitive to individual data points, potentially leading to overestimates or underestimates of variability. To address this issue, the Bootstrap method generates multiple resampled datasets from the original data, enabling the construction of a more accurate and stable estimate of the underlying data distribution. This can be observed in Fig. 8, where the distributions obtained with Bootstrap are narrower. Therefore, the non-Bootstrap distribution seems to be less representative of the true data characteristics, while the Bootstrap method provides a more reliable approach to extracting the data distribution, facilitating a more robust subsequent analysis.

### 4.4. Explainability of the proposed algorithm

To enhance the interpretability of our algorithm, we applied the MC-WindowSHAP algorithm, which is based on SHAP, a well-established method for interpreting ML models. Specifically, we divided our input signals into five time windows and applied the algorithm to each window individually. By leveraging MC-WindowSHAP, we can identify which latent dimensions contributed most significantly to positioning points within the latent space. Additionally, MC-WindowSHAP allowed us to determine which of the five windows had the most significant influence on the algorithm final decision regarding the presence or absence of TWA within $c_{\text{alt}}$.

**Fig. 9.**
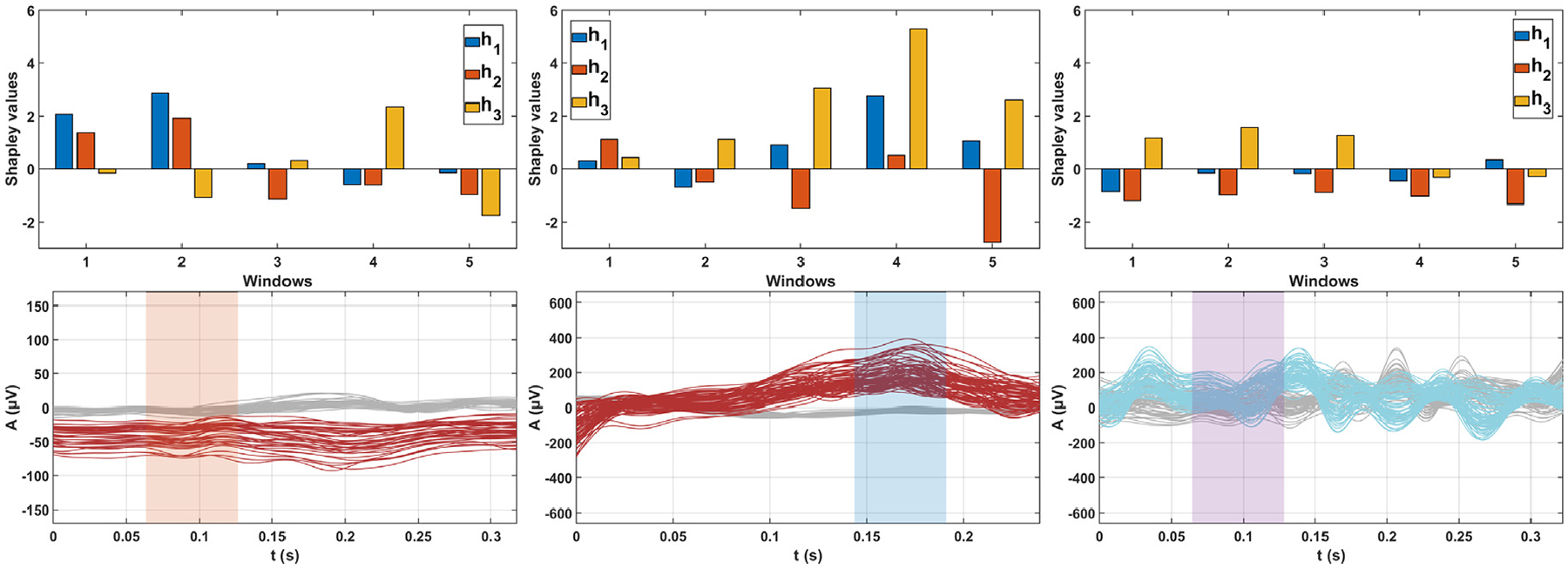
Shapley values for each latent space dimension and each window (top), with signals belonging to $c_{\text{alt}}$ highlighted in either red (when the method recognizes TWA) or blue (when the method does not recognize TWA), along with a set of randomly selected signals not belonging to $c_{\text{alt}}$ highlighted in gray (bottom). The results correspond to LQTS Patient 1 (left) and to ICM Patients 3 (middle) and 6 (right).

The top panels of Fig. 9 display the Shapley values for each latent dimension and each of the five windows. In contrast, the bottom panels show the signals belonging to $c_{\text{alt}}$, depicted either in blue if the MnL-based TWA detection algorithm detected no TWA or in red otherwise, and a set of randomly selected signals that did not belong to $c_{\text{alt}}$, depicted in gray. Specifically, results for LQTS Patient 1 and ICM Patients 3 and 6 are displayed from left to right. When applying MC-WindowSHAP to the signals belonging to $c_{\text{alt}}$ in LQTS Patient 1, it can be observed that the latent space dimensions of the greatest importance for the location of the points are $h_{1}$ in the first and second windows and $h_{3}$ in the fourth window (top, left panel in Fig. 9). In the latent space of LQTS Patient 1 (second row, first column in Fig. 6), $c_{\text{alt}}$ is clearly separated from the remaining communities, specifically located towards higher values of $h_{1}$ and slightly higher values of $h_{3}$ than the main cluster, which consistently aligns with the MC-WindowSHAP results. The window containing the latent dimension of highest importance was considered the one that most influenced the location of the signals with TWA in a specific region of the embedded space, and this window is highlighted in the lower panels of Fig. 9. For LQTS Patient 1 (bottom, left panel), it was the second window (highlighted in orange), closely followed by the first and fourth windows, as shown in the top panel. However, not only the samples in the second window but all signals belonging to $c_{\text{alt}}$ (red) are not centered at 0μV, as this patient exhibited amplitude-shifted TWA. In contrast, the signals not belonging to $c_{\text{alt}}$ (gray) are centered at 0μV. This clarified why our algorithm detected $c_{\text{alt}}$ as having TWA.

Regarding ICM Patient 3, $c_{\text{alt}}$ is not as clearly separated from the main cluster in the corresponding latent space (fourth row, third column in Fig. 6). Yet, our algorithm still identified $c_{\text{alt}}$ as exhibiting TWA. This can be explained by looking at Fig. 5 (fourth row, third column), which shows that this patient exhibits hump-shaped TWA in $c_{\text{alt}}$ signals. Fig. 9 (top, middle) indicates that latent dimension $h_{3}$ in the fourth window significantly influenced the positioning of the points with TWA in the embedded space. More generally, the highest Shapley values were concentrated in the third, fourth, and fifth windows, which capture the hump-shaped characteristics of the signals in $c_{\text{alt}}$, while the first two windows contributed minimally. Therefore, the hump-shaped features of the affected signals led our algorithm to conclude that $c_{\text{alt}}$ exhibited TWA.

Finally, ICM Patient 6 was identified by our algorithm as free of TWA. Examining the signals belonging to $c_{\text{alt}}$ for this patient (last row, second column in Fig. 5), we observe that they are not centered at 0μV. Instead, these signals are noisy and do not exhibit specific patterns such as hump-shaped or amplitude-shifted features. In Fig. 9 (top, right), it is evident that no particular window stands out as the main focus for our MnL-based TWA detection algorithm in determining whether $c_{\text{alt}}$ exhibited TWA, with the second window being the most relevant, closely followed by the others. In the bottom right panel, it can be observed that there are no significant differences between the signals belonging to $c_{\text{alt}}$ (blue) and those not belonging to $c_{\text{alt}}$ (gray). All the signals from this patient were extremely noisy, suggesting that the preprocessing stage was insufficient for this specific case, even though it was effective for the other sixteen subjects. This explains why our algorithm did not detect TWA in this patient.

Comparing all the panels in Fig. 9, we observe that the windows for ICM Patient 3 (middle) have the highest Shapley values, particularly those corresponding to the latter part of the signals. This is because the last windows primarily captured the TWA pattern, while the first two windows contributed less to the decision on the presence or absence of TWA, as they did not capture the TWA pattern. In contrast, this pattern of higher contributions was less evident in the other two cases. In LQTS Patient 1 (left), the TWA pattern was distributed across the entire signal, and in ICM Patient 6, the signals in $c_{\text{alt}}$ did not exhibit TWA. This interpretability analysis not only makes the model more explainable but also provides deeper insights into the temporal dynamics and latent variables influencing the classification of communities with TWA, thus enhancing the overall transparency of the model decision-making process.

### 4.5. Interpretation of results

When our method recognized TWA in $c_{\text{alt}}$, there appeared to be two different possibilities regarding the position of the ECG signals belonging to the community in the embedded space. In some cases, the target community was clearly separated from the rest of the communities, as indicated by the high distances between them, which was observed in control subjects with synthetically added TWA, in LQTS Patients 1 and 3, and in ICM Patient 1. This separation suggests the presence of a different community of ECG signals affected by TWA, which are sufficiently different from the remaining epicardial signals. In contrast, the unaffected signals were grouped in a different location within the lower-dimensional space, separated from where the ECG signals exhibiting alternans were located. On the other hand, there were instances where TWA was detected in $c_{\text{alt}}$ by our method, but the signals within $c_{\text{alt}}$ were not clearly separated from the rest, as shown by the lower distances between communities. This pattern was observed in LQTS Patients 4, 5, 6, and 8, as well as in ICM Patients 2, 3, 4, 5, and 7. In these cases, TWA seems to develop progressively in a specific epicardial area, with some signals within $c_{\text{alt}}$ exhibiting TWA to a lesser extent, explaining their proximity to the main manifold. Additionally, $c_{\text{alt}}$ was often located on the border of the main manifold. These two possibilities regarding the position of $c_{\text{alt}}$ in the embedded space may reflect different pathological patterns that could be associated with concordant and discordant alternans. These are two well-known TWA patterns in conventional ECG studies, each with different clinical implications (You et al., 2021). In concordant alternans, the amplitude or morphology of T-wave changes is consistent across multiple leads, suggesting that TWA affects a large, specific cardiac region uniformly. This uniform pattern could indicate more gradual and progressive changes in cardiac tissue, which might be represented in the latent space generated by our algorithm as $c_{\text{alt}}$ being located close to the main manifold, reflecting progressive differences between signals with and without TWA. In contrast, discordant alternans involve T-wave variations that differ between leads, indicating a lack of synchronization across different heart regions. This pattern may be linked to TWA in smaller epicardial areas and could appear suddenly rather than progressively. In the latent space, this might be represented by points far from the main manifold, suggesting localized disturbances, such as scarring. To draw more meaningful conclusions about our method ability to differentiate between these TWA patterns, a more targeted study is needed, which would enhance its application in clinical diagnostics.

## 5. Discussion

In this study, the electrophysiologic substrate in patients with LQTS and ICM was characterized using noninvasive ECGI, which can map the ventricular epicardium with high spatial resolution and allow the localization of areas with ventricular repolarization heterogeneities. We used TWA as a biomarker for abnormal electrophysiology and arrhythmias to localize such regions. The gold standard for detecting TWA is Holter monitoring, but this test does not provide spatial information about the characteristics of TWA as ECGI does. ECGI allows us to investigate the location where the biomarker is more prominent, the size of the affected area, and even different types of TWA patterns. In recent years, body surface ECG has been employed to measure markers for ventricular repolarization in patients with LQTS and ICM. However, the spatial resolution of conventional ECG studies is limited, resulting in a lack of sensitivity and specificity in the results (Vijayakumar et al., 2014; Cuculich et al., 2011). This limitation can be addressed using ECGI, which offers a higher spatial resolution of cardiac electrical activity.

However, the advantage that ECGI provides higher spatial resolution also presents an issue due to the difficulties in simultaneously analyzing such a large number of ECG signals. This is where MnL techniques come into play. These techniques have previously been used for data visualization when original data dimensions prevented us from understanding the data. It is worth considering that the embedded space they create mimics the original space of characteristics with high precision. In particular, UMAP is known for maintaining data structure at different levels. UMAP considers the spatiotemporal information in ECGI data simultaneously and projects similar ECG signals together in the latent space, facilitating the subsequent analysis of TWA. Therefore, given that the lower-dimensional space can describe the original space with accuracy, working in this new space allows for the identification of relevant patterns of TWA that cannot be seen in the original space due to the overwhelming quantity of signals (high dimension in the spatial domain) and their duration in time, which can be greater than one minute in some cases (high dimension in the temporal domain).

The novel TWA identification method proposed in this paper has shown a new way for biomarker automatic design. Our algorithm achieved the highest possible success rate in cases with known solutions, i.e., control subjects who did not exhibit TWA, synthetic patients with synthetic TWA, and control subjects with synthetic TWA. While identifying TWA in synthetic patients with added synthetic TWA is straightforward, detecting TWA in control subjects with added synthetic TWA is as challenging as in actual patients due to intrinsic noise levels that can never be eliminated entirely. In general, our MnL-based algorithm interpretability can be justified by observing the embedded space and the input signals, which additionally showed different kinds of TWA manifestations. Using a community detection algorithm, signals with similar characteristics are clustered into the same group, allowing us to analyze each cluster separately afterward. Identifying the cluster with more variations between even and odd T-waves enabled the analysis of the shape of the TWA signals obtained by subtracting even and odd T-waves. Through this analysis, we identified different types of TWA, namely hump-shaped TWA and amplitude-shifted TWA, although they also appeared mixed up in some cases. The former type consists of morphological differences between consecutive even and odd T-waves, while the latter refers to changes in amplitude between consecutive even and odd T-waves. In all cases, the ECG signals with any of these kinds of TWA were identified by the algorithm, and those communities with signals that did not have differences between their T-waves were identified as healthy. Specifically, in cases where the method did not detect TWA, the mean signal was centered at 0μV, it barely varied over time, and all the signals in the community behaved similarly to the mean signal and each other. Conversely, in cases where the algorithm identified TWA, the mean signal was either hump-shaped or not centered at 0μV, or signals in the community behaved considerably differently from the others, exhibiting either of the two described patterns. This analysis enabled us to understand the rationale behind every decision made by our algorithm, confirming its coherence and validity. Moreover, the decision-making process of our algorithm relies on a statistical test, namely Bootstrap resampling. Visualizing data distributions alongside extracting CIs provided valuable insights into algorithm reasoning, facilitating a deeper understanding of the decision-making mechanism. Our findings align with the insights presented in Cuculich et al. (2011) and Vijayakumar et al. (2014). In the former, the authors characterized the scar tissue generated in patients with ICM, examining its location, size, and morphology. In the latter, the focus was on ventricular activation, recovery times, activation-recovery intervals, and repolarization dispersion in patients with LQTS. In contrast, our study analyzed T-waves and their beat-to-beat variations in amplitude, morphology, or both, in patients with ICM or LQTS. Despite focusing on different ventricular electrophysiological substrates, all three studies converge on the conclusion that the spatial characterization of repolarization heterogeneities in the human ventricles can be highly informative and can provide critical insights into the mechanisms underlying certain arrhythmias.

### ECG Preprocessing Techniques

Efficient preprocessing of ECG signals is necessary for accurately analyzing TWA, as it consists of subtle variations that may be mistaken for noise. Therefore, the design of filters is fundamental to ensure that physiological information related to TWA is preserved, along with the characteristics of the T-wave, which manifests as a low-frequency wave in ECG recordings. There exist numerous studies that focus on the ECG signal preprocessing, and different filters have been proposed in the literature to remove noise while retaining information about the cardiac electrical activity. In Rashid et al. (2011) and Ahmed et al. (2023), the authors developed and tested specialized filters for baseline wandering. Both the PPGAA presented in Rashid et al. (2011) and the InvBase presented in Ahmed et al. (2023) showed promising results, outperforming traditional methods for baseline wandering in ECG and EEG signals, respectively. For high-frequency noise removal, in Krishnamurthy et al. (2015), the authors comprehensively compared various filter types, concluding that finite impulse response filters outperform infinite impulse response filters. Similarly, in Pandey and Giri (2016), the authors introduced a novel high-frequency noise filter combining a moving average filter and a polynomial curve-fitting algorithm, demonstrating both accuracy and computational efficiency. In this work, we applied a spline detrending filter to remove baseline noise and a low-pass Butterworth filter with a 30 Hz cutoff frequency to eliminate high-frequency noise. Both filters were carefully designed to avoid removing physiological or pathological information related to the T-wave, which is the primary focus for estimating TWA. Additionally, we demonstrated the effect of these filters on different ECG signals, confirming that T-waves remained unaffected after noise removal.

### MnL Approach and Interpretable AI.

As previously stated, UMAP is designed to preserve both the global and local structure of the input data when projecting it into a lower-dimensional space. In our specific case, this means that signals similar to each other over time will be located close to one another in the latent space. This occurs because the graph representing the lower-dimensional relationships of the data is generated in a way that mimics the graph of relationships in the higher-dimensional space. UMAP minimizes the differences between these two graphs, thus preserving both linear and non-linear relationships in the latent space. UMAP focuses on retaining essential characteristics of the signals, and the interpretability algorithm we used, MC-WindowSHAP, helps identify which windows UMAP primarily focuses on when projecting the input data into specific regions of the latent space, further enhancing the explainability of our proposed method. For example, when the TWA pattern is hump-shaped, UMAP focuses on the signal windows that capture this distinctive shape while giving minimal attention to the rest of the windows. In contrast, when the TWA pattern manifests as an amplitude shift, UMAP distributes focus across all windows, as this pattern is consistent throughout the entire signal and does not appear in isolated segments. A similar observation is made when no TWA is present. In those cases, there are no significantly higher Shapley values for any specific window, indicating that any part of the signal can contribute equally to determining the absence of TWA. Although UMAP tries to preserve as much relevant information from the original high-dimensional data as possible, specific fine-grained details may not be retained in the lower-dimensional space. This is because the latent space representation is a compressed version of the original data. However, not only are some subtle details potentially lost, but redundant information is also reduced in the lower-dimensional representation, which can enhance the identification of clusters based on essential features of the data.

The Louvain algorithm focuses on the topology of the lower-dimensional graph generated by UMAP, i.e., the structural arrangement of the data points and the connections among them in the embedded space. Said algorithm groups data points into communities based on their relationships within the lower-dimensional graph. Since the algorithm works on the lower-dimensional graph created by UMAP, it inherently captures the relevant features of the original signals that UMAP preserved in its latent space. This means that lower-dimensional data points corresponding to signals with hump-shaped TWA contain information specifically about that pattern. In contrast, data points corresponding to amplitude-shifted TWA capture information about the amplitude shift. Similarly, data points for signals without TWA contain more general information about the signals, as revealed by MC-WindowSHAP. The Louvain algorithm generates communities by grouping signals that are similar to one another, as reflected in the latent space. For instance, signals exhibiting a hump-shaped TWA pattern are grouped into the same community because UMAP preserves the features representing this pattern in the lower-dimensional space, which the Louvain algorithm then uses for community detection. Therefore, we can understand a community as a set of signals that share common characteristics that UMAP has retained in its lower-dimensional representation.

### Known-solution Cases in ECGI Algorithm Design.

In this study, we incorporated four synthetic patients as part of the validation process for our TWA detection method. Two of these synthetic patients were designed as idealized examples where the location of TWA was easily recognizable by any detection method. These subject data did not resemble actual subject data and were explicitly included to verify the basic functionality of our algorithm. Creating fully controlled scenarios ensures the algorithm operates as intended and can successfully identify evident alternans patterns. On the other hand, the other two synthetic subjects were more complex. While synthetic alternans were introduced into these cases, their ECG signals were derived from actual patient data, specifically, control-subject data. As a result, these recordings contained real-world characteristics, such as noise and other artifacts commonly found in real ECG signals. This made these synthetic cases more challenging, as they closely resembled actual clinical conditions. The alternans signal amplitude in these cases was adjusted based on the findings of a previous study (Sanchez-Carballo et al., 2024), in which our group determined the minimum amount of TWA required for conventional TWA estimation methods to identify TWA in control subjects. These more realistic synthetic cases provided a valuable test of our algorithm robustness. Specifically, they allowed us to demonstrate that our method performs at least as well as conventional TWA estimation techniques, even under noisy and challenging conditions. Although these synthetic patients do not fully replicate the variability and complexity of real subjects, they play an essential role in assessing the initial performance of our algorithm.

### Comparison with Other Recent TWA Detection Algorithms.

There exist other works also focusing on improving existing TWA estimation methods and developing new approaches. For example, in Ullah et al. (2020), the authors addressed the challenge of estimating TWA from stress tests, which generate particularly noisy ECG signals. They proposed the GDTF, which involved three stages: ECG signal preprocessing, data reduction, and TWA analysis using hypothesis testing. In the data reduction stage of their approach, the authors aimed to minimize data redundancies by eliminating inefficient characteristics. For TWA analysis, they employed probabilistic and statistical signal models that use statistical parameters to determine the presence or absence of alternans through hypothesis testing. However, as described in the original paper, this method reported good results at a very high computational cost. Our work also includes a data reduction step before determining the presence of TWA. However, we use UMAP instead, which has been shown to generate a manifold that preserves both the global and local structure of the original data. Rather than relying on statistical and probabilistic algorithms, our approach takes advantage of UMAP ability to retain essential characteristics while projecting data into a lower-dimensional space. This allows similar signals to cluster together, enabling the Louvain algorithm to identify specific communities and assess their nature. Our method then applies hypothesis testing theory similarly to GDTF. However, instead of focusing on statistical parameters, we use Bootstrap resampling to analyze the distances between communities in the embedded space. Additionally, our algorithm is computationally efficient, with execution times of approximately 1.5 min for a mesh containing 599 nodes when run on MATLAB 2022b, on an 11th Gen Intel(R) Core(TM) i7-11800H @ 2.30 GHz and a 64-bit operating system.

Another study focusing on developing a novel TWA detection method is Ullah et al. (2022), in which the authors introduced the PGLM approach for TWA detection in noisy environments. This algorithm addressed the non-linear and non-stationary nature of ECG signals by using first-order piecewise polynomials with cubic spline interpolation. Then, the coefficients were evaluated using the least squares method. The PGLM was compared with other TWA estimation methodologies, including the modified moving average method, which is particularly accurate in noisy settings. This approach differs from ours, as rather than fitting the alternans signal to a specific curve, our method focuses on extracting particular characteristics from the ECG signals to identify features associated with TWA. Additionally, while our algorithm employs hypothesis testing to determine the presence or absence of TWA, the approach in Ullah et al. (2022) provides a TWA alternans signal without making a definitive decision. Furthermore, PGLM is designed explicitly for noisy ECG signals, whereas our method is tailored to ECGI data, which is typically collected in less noisy environments.

In Pascual-Sánchez et al. (2024), the focus was also on investigating alternative methods for TWA detection. In this work, the authors compared a conventional TWA estimation method, precisely the spectral method, with two widely known ML techniques, K-nearest neighbors and random forest. This study also focused on feature extraction, which is similar to our approach, and also provided a decision on the presence or absence of TWA. However, rather than solving this binary classification problem through hypothesis testing, they used ML algorithms directly as we do. They reported positive results with both ML methods, achieving accuracy values above 0.85 on the test set and obtaining better outcomes than the spectral method. In contrast, our method employs MnL for feature extraction, which enables us to visualize intermediate results and enhances the explainability of our final decisions.

### On the Statistical and Spatiotemporal Insights.

The general objective of Ullah et al. (2020, 2022) and Pascual-Sánchez et al. (2024) and our study was to develop new methods for TWA estimation. However, several key differences distinguish our study from the others, contributing to its novelty. First, our algorithm evaluated TWA from a spatiotemporal perspective based on the hypothesis that alternans are more pronounced in certain cardiac regions. Second, our algorithm provided interpretable results by allowing the visualization of intermediate steps. Each point in the latent space corresponded to an alternans signal that could be visualized. Moreover, the interpretability of the proposed method was enhanced by the application of the MC-WindowSHAP algorithm, which identified the ECG signal segments that most influenced the positioning of data points in the lower-dimensional space. These points make our work the first to propose a TWA estimation method tailored explicitly to ECGI, providing interpretability that offers valuable insights into the spatial and temporal dynamics of TWA.

The results of our proposed method highlight the potential of leveraging both temporal and spatial information from ECGI to improve TWA estimation and localize areas of the epicardium with more pronounced TWA. Unlike existing methods, which analyze each ECG signal independently, our MnL approach incorporates the full spectrum of information available in ECGI, capturing relationships between signals in both time and space. This represents a significant advance in TWA estimation, allowing for a more comprehensive analysis of the electrical activity across the heart. Moving beyond the traditional analysis applied to conventional ECG data, our method offers a more accurate and detailed understanding of TWA manifestations. Furthermore, while recent ML-based methods for TWA estimation have shown promise, as exemplified by Pascual-Sánchez et al. (2024), they predominantly rely on supervised learning models that require large datasets of labeled ECG signals. These methods focus on learning generalized alternans patterns applicable across different subjects, but individual variability can limit their performance. Our approach, by contrast, is subject-specific and unsupervised, enabling personalized TWA detection. By leveraging the rich spatial and temporal data from ECGI, our method differentiates between TWA and non-TWA signals within the same individual, offering a tailored estimation process that does not depend on extensive labeled data.

### Study Scope and Limitations.

Despite the advantages of our novel TWA identification technique, there is room for improvement. One limitation of this study is the small sample size, consisting of 17 real subjects, which may affect the generalization capacity of our findings. However, due to the lack of open-access datasets containing real ECGI data from patients exhibiting TWA, obtaining a larger cohort was not feasible at this stage. This limitation may occur due to the novelty of the imaging modality, which results in a limited number of studies addressing the problem we are dealing with using this type of data. To mitigate this limitation, we generated synthetic patients according to Sanchez-Carballo et al. (2024) to evaluate the method under controlled scenarios. Moreover, the data used in this study have been analyzed in other works (Cuculich et al., 2011; Vijayakumar et al., 2014), providing meaningful insights. As a pilot study, these results offer crucial preliminary evidence, but future studies should aim to expand the dataset to validate these findings further.

Our approach involved using UMAP for dimensionality reduction and the Louvain algorithm for community detection, applied independently to each subject data. Our goal was not to generalize findings across different individuals but to identify specific patterns within the data of a single subject. This subject-specific model was designed to capture unique and potentially hidden personal features by treating each participant’s data independently. While the small sample size is a recognized limitation, the subject-specific nature of our analysis helps mitigate this issue. Since the analysis is tailored to each individual, the risk of overfitting in the traditional sense is not a concern. Overfitting usually involves a model learning patterns specific to a training dataset, which reduces its ability to generalize to unseen data. However, our method is not intended to predict new or unseen data. Instead, it focuses on extracting patterns unique to each individual case. By analyzing each subject’s data independently, we avoid the pitfalls of generalizing to a broader population, which can be particularly challenging with a small sample size. UMAP and the Louvain algorithm help reduce data complexity while preserving the inherent structure, enabling us to detect meaningful communities within each subject signals. This approach is crucial for understanding subject-specific dynamics, which can vary significantly between individuals. Although a larger sample size would undoubtedly enhance the robustness of our findings, the personalized nature of our analysis provides valuable insights into each individual unique characteristics. Consequently, our study was designed not to create a universal model but to capture the specific features of each participant’s data.

By leveraging MnL, community detection, and Bootstrap resampling, we have developed the first TWA detection algorithm tailored explicitly for ECGI data. UMAP captures complex patterns in high-dimensional ECGI data, while the Louvain algorithm effectively identifies the cluster of signals that may exhibit TWA. Bootstrap resampling further enhances the robustness of our algorithm by ensuring decisions are grounded in statistical reliability. Our algorithm has demonstrated a remarkable success rate, accurately detecting TWA in all patients with known outcomes. Additionally, the algorithm decisions align with expectations when analyzing the input signals, which enhances its interpretability. To further strengthen explainability, we have applied the MC-WindowSHAP interpretability algorithm, which is customized for our specific data type. MC-WindowSHAP highlights the specific areas of the input signal that the algorithm focuses on to determine the presence or absence of TWA within a given community. This transparency may allow clinicians to understand better how the algorithm arrives at its conclusions, thus increasing trust in the decision-making process.

## 6. Conclusion

This work presents an innovative algorithm for detecting TWA using ECGI. Apart from being the first TWA estimation method tailored to ECGI, it is also the first methodology capable of non-invasively identifying specific areas in the epicardium affected by TWA. Our method uses UMAP to reduce the input data dimensions, the Louvain algorithm to identify communities with clinically relevant characteristics, and a Bootstrap-based TWA detector to determine whether the subject exhibits TWA statistically. The dimensionality to which UMAP reduces the original data was analyzed. Reducing the data to eighteen dimensions was identified as the optimal choice, as it provided the greatest separation between the community with TWA and the healthy signals. This configuration yielded an average normalized distance of 0.28 between the two groups, compared to 0.26 when the data was reduced to fifteen dimensions, which resulted in the next highest average normalized distance. Additionally, we developed and applied the MC-WindowSHAP to enhance the algorithm interpretability. This interpretability algorithm identifies the segments of the original ECG signals on which UMAP focuses to generate the embedded space and the ECG segments that most affect the algorithm decision. Our algorithm achieved the maximum success rate in the six cases with known outcomes in our database, including two control subjects without TWA, two synthetic subjects with TWA that any estimation technique could easily recognize, and two control subjects with added TWA, which contain the inherent noise characteristic of real recordings but still represent near-real cases. Our algorithm also reached coherent conclusions for patients with LQTS and ICM, according to the visualization of input signals belonging to the TWA-dominant community. These results open new avenues for investigating TWA. In the future, examining the algorithm behavior in more extensive datasets containing labeled real-world ECGI data would be beneficial, as this would increase the quality and robustness of the validation process. Once the algorithm has been rigorously assessed with more real patient data, exploring its clinical applicability for arrhythmia detection would be interesting.

## Figures and Tables

**Table 1:** Number of nodes, ECG signals duration, T-waves duration, and the number of beats in ECG signals for all subjects in our dataset. The units for duration measurements are in seconds.

	Number of nodes	Signal duration	T-Wave duration	Number of beats
C1	1002	65.38	0.28	65
C2	1002	36.00	0.28	29
Synth. C1 + TWA	1002	21.48	0.27	20
Synth. C2 + TWA	1002	24.41	0.28	20
C1 + TWA	1002	65.38	0.28	65
C2 + TWA	1002	36.00	0.28	29
LQTS1	502	60.00	0.31	66
LQTS2	502	62.00	0.40	50
LQTS3	502	62.00	0.40	60
LQTS4	502	35.30	0.39	26
LQTS5	502	60.00	0.31	56
LQTS6	502	62.00	0.31	58
LQTS7	502	60.00	0.25	58
LQTS8	502	60.00	0.36	56
ICM1	953	9.26	0.22	9
ICM2	734	5.59	0.29	5
ICM3	752	9.59	0.24	14
ICM4	810	10.49	0.33	10
ICM5	502	69.48	0.15	128
ICM6	1054	9.76	0.32	8
ICM7	1002	9.76	0.26	10

**Table 2:** Normalized distances between $c_{\text{alt}}$ and the other points in the latent space. The average normalized distances for each dimension are included in the last row. For each patient, bolded and italicized values are the highest and second highest, respectively.

	3	6	9	12	15	18	21
C1	0.1344	0.1759	0.1722	0.1670	0.1684	0.1873	0.1720
C2	0.2119	0.1886	0.1513	0.2142	0.2077	0.1519	0.2050
Synth. C1 + TWA	0.7005	0.5851	0.7618	0.8349	**0.9769**	0.8750	*0.8932*
Synth. C2 + TWA	0.7362	0.6613	0.7424	0.7779	**0.8414**	*0.8012*	0.7010
C1 + TWA	0.5251	0.4459	0.5122	0.6200	0.7154	*0.7618*	**0.8209**
C2 + TWA	0.4088	0.5523	0.6524	0.7015	*0.7100*	**0.7967**	0.6257
LQTS1	0.2616	0.3184	0.3112	0.3054	**0.3735**	*0.3549*	0.3003
LQTS2	0.1793	**0.2448**	0.1883	0.1937	0.2043	0.2011	*0.2044*
LQTS3	0.1752	0.1755	0.1816	0.1797	0.1853	**0.1957**	*0.1856*
LQTS4	0.1365	0.1849	**0.1987**	0.1350	0.1293	*0.1983*	0.1636
LQTS5	0.1320	0.1652	*0.1960*	**0.3033**	0.1826	0.1817	0.1546
LQTS6	0.1576	0.1770	0.1615	0.1728	**0.2062**	*0.2043*	0.1875
LQTS7	0.2027	0.1913	0.1899	*0.2167*	0.1997	**0.2353**	0.2151
LQTS8	0.2044	0.2067	0.1955	0.2153	*0.2398*	**0.2400**	0.2063
ICM1	0.1178	0.1212	0.1314	*0.1479*	0.1357	**0.1736**	0.1471
ICM2	0.1564	0.1692	**0.1975**	0.1597	0.1889	*0.1922*	0.1651
ICM3	0.1589	*0.2226*	0.2133	**0.2282**	0.2218	0.2068	0.1961
ICM4	0.1050	**0.2501**	0.1320	0.1228	0.1877	*0.2415*	0.1274
ICM5	0.1540	0.1866	0.2004	0.1978	0.1888	*0.2117*	**0.2197**
ICM6	0.1424	0.1346	0.1327	**0.1585**	*0.1542*	0.1427	0.1325
ICM7	**0.2542**	0.1642	0.1476	0.1846	*0.1961*	0.1736	0.1951
Average	0.2042	0.2300	0.2313	0.2496	*0.2600*	**0.2772**	0.2498

**Table 3:** Decision on the presence or absence of TWA, according to the Bootstrap-based proposed method.

C1	No	C1 + TWA	Yes	LQTS3	Yes	LQTS7	No	ICM2	Yes	ICM5	Yes
C2	No	C2 + TWA	Yes	LQTS4	Yes	LQTS8	Yes	ICM3	Yes	ICM6	No
Synth. C1 + TWA	Yes	LQTS1	Yes	LQTS5	Yes	ICM1	Yes	ICM4	Yes	ICM7	Yes
Synth. C2 + TWA	Yes	LQTS2	No	LQTS6	Yes						

## Data Availability

The source code used in this study is publicly available and can be accessed at https://github.com/estelasc/TWA-analysis-toolbox. The repository contains full implementations of the methods described in this work to enhance reproducibility and facilitate understanding of the analyses performed.

## Data availability

The authors do not have permission to share data.
